# Cadmium exposure modulates the gut-liver axis in an Alzheimer’s disease mouse model

**DOI:** 10.1038/s42003-021-02898-1

**Published:** 2021-12-15

**Authors:** Angela Zhang, Megumi Matsushita, Liang Zhang, Hao Wang, Xiaojian Shi, Haiwei Gu, Zhengui Xia, Julia Yue Cui

**Affiliations:** 1 Department of Environmental and Occupational Health Sciences, University of Washington, Seattle, WA USA; 2 Arizona Metabolomics Laboratory, College of Health Solutions, Arizona State University, Phoenix, AZ USA

**Keywords:** Biomarkers, Predictive markers, Systems biology

## Abstract

The human Apolipoprotein E4 (ApoE4) variant is the strongest known genetic risk factor for Alzheimer’s disease (AD). Cadmium (Cd) has been shown to impair learning and memory at a greater extent in humanized ApoE4 knock-in (ApoE4-KI) mice as compared to ApoE3 (common allele)-KI mice. Here, we determined how cadmium interacts with ApoE4 gene variants to modify the gut-liver axis. Large intestinal content bacterial 16S rDNA sequencing, serum lipid metabolomics, and hepatic transcriptomics were analyzed in ApoE3- and ApoE4-KI mice orally exposed to vehicle, a low dose, or a high dose of Cd in drinking water. ApoE4-KI males had the most prominent changes in their gut microbiota, as well as a predicted down-regulation of many essential microbial pathways involved in nutrient and energy homeostasis. In the host liver, cadmium-exposed ApoE4-KI males had the most differentially regulated pathways; specifically, there was enrichment in several pathways involved in platelet activation and drug metabolism. In conclusion, Cd exposure profoundly modified the gut-liver axis in the most susceptible mouse strain to neurological damage namely the ApoE4-KI males, evidenced by an increase in microbial AD biomarkers, reduction in energy supply-related pathways in gut and blood, and an increase in hepatic pathways involved in inflammation and xenobiotic biotransformation.

## Abstract (web summary)

Angela Zhang et al. report that humanized Apolipoprotein E4 (ApoE4) knock in mice exposed to cadmium had prominent Alzheimer’s Disease-related changes in the makeup of their gut microbiota. They ultimately identify a host genetic-gut microbiome axis susceptible to environmental cadmium insult that may serve as a strong facilitator in cognitive and energy homeostasis.

## Introduction

Alzheimer’s disease (AD) is a progressive neurodegenerative disease that is characterized by the degeneration of neurons that are involved in memory, which progressively leads to continuous decline in thinking, language, behavior, and social skills. With a growing aging population, this disease is an increasingly important public health problem. In 2016, it was estimated that 5.4 million Americans had AD; by 2050, this number is projected to be 13.8 million. Although funding for AD has increased in recent years, the etiology of this neurodegenerative disease is complex and still unclear^1^.

Apolipoprotein (ApoE) is a protein that binds to lipids for transport and is thought to be critical for healthy brain function^2^. The ε4 variant (ApoE4), which has a worldwide frequency of 13.7%, is the strongest known genetic risk for AD^3^. However, this variant does not necessarily lead to the development of AD, suggesting that other risk factors must interact with ApoE4 to explain the etiology of AD. Cadmium (Cd), which can be found in sources such as rice grown in Cd-laced water, is a toxic heavy metal that has a long biological half-life due to its slow excretion from the body. There is accumulating evidence that Cd is a neuro-toxicant that may lead to AD-like neurodegenerative disorders. For example, metallothionein-3 (MT-III), which plays an important role in Cd excretion, has been found to be deficient in AD patients^3^. In order to recapitulate the effects of the human genetic polymorphism of the ApoE allele in vivo, the humanized ApoE-knock in (KI) mice were generated with either the human ApoE4 (susceptible allele) or ApoE3 (common allele), thus replacing the ApoE gene in mice^4^.

The ApoE4-Cd gene-environment interaction was explored in our recent study^5^. Specifically, through 14-weeks of Cd exposure via drinking water at a concentration (0.6 mg/L per day) that resulted in a blood Cd concentration relevant to the general human blood Cd concentrations, it was shown that ApoE4 knock-in (ApoE4-KI) mice had greater deficits in cognitive function and decreased adult hippocampal neurogenesis compared to ApoE3-KI mice. Furthermore, male mice were more susceptible to this gene-environment interaction effect and showed earlier cognitive deficits than females^5^. This study provided direct evidence that ApoE4-KI mice (and especially males) are more vulnerable to the toxic effects of Cd.

The gut-brain axis has been established as an important communication pathway between the central nervous system (CNS) and the gut microbiome^6^. There is a large body of evidence connecting AD with changes in the microbiome and there has been increasing motivation to understand how the host genes and the function microbiome interact with each other within the pathogenesis of AD^7,8^. Within APP/PS1 mice, a transgenic AD mouse model which have elevated production of β-amyloid, germ-free (GF) mice had a drastic reduction of cerebral β-amyloid pathology, whereas increased cerebral Aβ amyloid pathology was observed in GF mice colonized with the “diseased” microbiota of conventional mice^9^. In humans, AD patients had decreased gut microbial diversity which was compositionally distinct from age- and gender-matched individuals from healthy controls^10^. Specifically, patients with AD had an increased abundance of pro-inflammatory *Escherichia/Shigella* and decreased abundance of anti-inflammatory *Eubacterium rectale* in their intestines^11^.

Xenobiotic transformation plays a fundamental role in drug metabolism. Cytochrome P450s, an important family of Phase I oxidation enzymes, are responsible for the metabolism of almost 90% of all drugs. Phase 2 enzymes, such as those involved in conjugation reactions, are responsible for increasing the polarity of drug molecule. The resulting product is more water soluble and can be readily excreted out of the system by transporters^12^. It is important to note that many AD patients take multiple therapeutic drugs for the management of AD and a number of other complex co-existing diseases^13^. In monogenic studies, APOE4 genotype carriers are the worst responders to conventional treatments, and certain cholinesterase inhibitors used to treat AD require P450s to be metabolized^13^.

Liver and intestine are major organs for xenobiotic biotransformation and nutrient homeostasis. The pharmacological response in AD depends on the interaction of genes involved in xenobiotic biotransformation as well as factors associated with AD pathogenesis^13^. Previously, it has been shown that the APP/PS1 AD mouse model had altered expression of hepatic drug-metabolizing enzymes and small intestinal drug transporters^14^. In APP/PS1 mice, Cyp51a1 and Cyp2c29 proteins were upregulated in liver, whereas the phase-II conjugation enzyme UDP-glucuronosyltransferase (Ugt2b5) and the efflux transporter multidrug resistance-associated protein (Mrp2) were upregulated but the monocarboxylate transporter (Mct1) was downregulated in intestine^14^. Elevated hepatic Cyp (Cyp2b, 2e1, 3a, and 4a) activity has been observed in the Tg2575 AD mouse model^15^. Furthermore, liver dysfunction has been suggested as a novel player in AD progression, evidenced by dysregulation in hepatic lipid metabolism and chronic inflammation^16^. In addition, an elevation in the ratio of aspartate aminotransferase to alanine aminotransferase, which are biomarkers for liver injury, were associated with AD diagnosis, increased amyloid-beta deposition, and reduced brain glucose metabolism^17^. Dysregulation of drug processing genes caused by Cd toxicity may lead to adverse drug-drug interactions.

Cd modulates xenobiotic biotransformation within the liver in various animal models. For example, a single intraperitoneal (i.p.) dose of Cd (2 mg/kg) to rats acutely reduced the hepatic microsomal drug metabolism activity both in vivo and in vitro^18^. Acute Cd exposure via i.p. (0.84 mg/kg) and per os (p.o.) (>80 mg/kg) has also been shown to inhibit the hepatic microsomal metabolism of hexobarbital and aniline in male rats^19^. It has been suggested that Cd converts cytochrome P450s (Cyps) to P420s in rat liver microbiomes, and such destruction of Cyps contributes to the reduction of the drug metabolism pathways^20^. Both sex and age can modify susceptibility to cadmium-induced decline in hepatic drug metabolism pathways: male rats are more sensitive to Cd than females, whereas older rats are more sensitive to Cd than younger ones^20^. Another study showed that an acute testicular toxic dose of Cd (2.0 mg/kg i.p) reduced hepatic microsomal aryl hydrocarbon hydroxylase (now known as Cyp1a) and aminopyrine N-demethylase (now known as Cyp3a) activities more in male rats than in female rats in a testosterone-dependent manner^21^. In the intestine of mice, acute Cd exposure activated the xenobiotic-sensing nuclear receptor constitutive androstane receptor (CAR) and upregulated its target gene Cyp2b10^22^. However, chronic Cd exposure in drinking water has not been shown to produce a significant effect on hepatic drug metabolism (up to 200 mg/L and up to 12 weeks)^18,19^. It has been suggested that the induction of metallothionein, which binds Cd and facilitates its export out of the liver, may render such tolerance^19,23^.

Apart from alterations in drug metabolism, acute Cd exposure has been shown to perturb the expression of genes involved in oxidative stress, DNA damage, cell cycle, and inflammatory response and produce reactive oxygen species (ROS), leading to DNA strand breaks and lipid peroxidation in rat liver^24^, as well as decreased gap junction intercellular communications in mouse liver^25^. In addition, there is an interplay between Cd-mediated hepato-toxicity and hepatic energy metabolism. For example, in rat models, it has been shown that diabetes exacerbates Cd-induced liver toxicity, whereas insulin therapy is beneficial in restoring liver function^26^. Ten-week exposure to Cd (10 mg/L) in drinking water increased hepatic triacylglycerol, serum free fatty acid, and triglyceride levels, and increased the mRNAs of genes involved in de novo free fatty acid synthesis and transport as well as triglyceride synthesis in the mouse liver^27^. In addition, this study also showed that Cd exposure modulates the gut microbiome in mice, evidenced by the reduction of *Firmicutes* and *γ-proteobacteria*, and it was hypothesized that this may increase serum lipopolysaccharide and induce hepatic inflammation, which will in turn perturb hepatic energy homeostasis^27^.

Although Cd and AD each have been shown to impact the xenobiotic and energy metabolism of liver and other metabolic organs, very little is known regarding to the extent that cadmium and the ApoE genotype interact to modulate the xenobiotic and intermediary metabolism pathways within the gut-liver axis, and how changes in the hepatic gene expression signatures correspond to the gut dysbiosis and neurodegenerative phenotypes.

In order to fill this critical knowledge gap, we used humanized homozygous ApoE3-KI and ApoE4-KI mice to elucidate the effect of Cd exposure on the gut-liver axis and sought to unveil potential peripheral biomarkers for and/or contributors to the susceptibility to Cd-mediated neurotoxicity between sexes and the ApoE genotypes. Specifically, we characterized the interaction between Cd and the gut microbiome in an AD mouse model through: (1) identifying significantly involved bacterial taxa in the gut microbiota and microbial metabolites and (2) characterizing perturbations in the host hepatic transcriptome especially the genes involved in drug metabolism, transport, and inflammation.

## Results

**Fig. 1:**
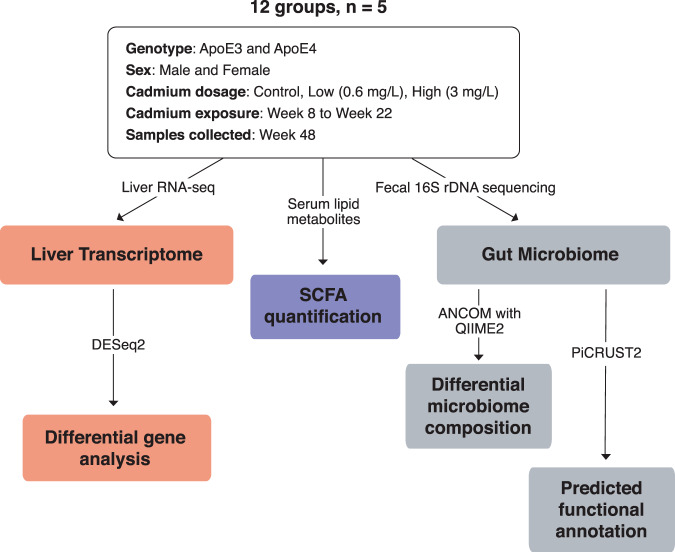
Workflow. Workflow showing the experimental design of the study.

The workflow of the experiment is outlined in Fig. 1.

### Diversity measures

**Fig. 2:**
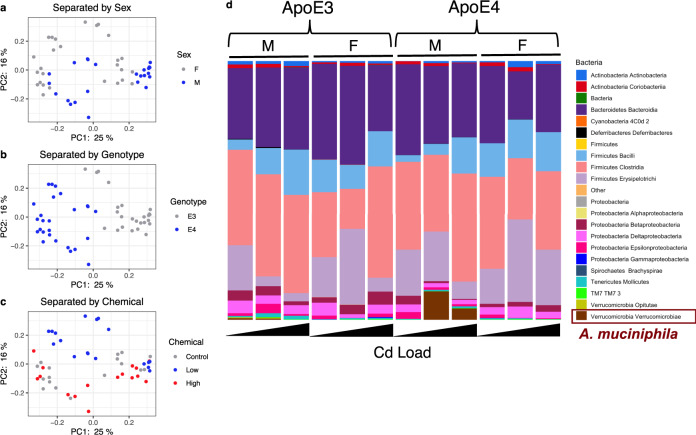
Beta diversity separated by sex, genotype, and chemical effects. Principal coordinates analysis (PCoA) using the Bray-Curtis dissimilarity measure separated by **a** Male and Female; **b** ApoE3 and ApoE4; and **c** Control, Low Cd and High Cd doses. **d** Class**-**level stacked bar plots between groups separated by sex, genotype, and chemical exposure; Stacked bar plots indicate the average taxonomic level 3 (up to class) proportion across sex, genotype and chemical exposure. Data were analyzed using QIIME 2 as described in “Methods”.

The number of reads sequenced and the mapping statistics of the 16S rDNA sequencing data are shown in Supplementary Table 1. In groups with the same genotype and sex, there were no marked differences in alpha diversity among control, low and high doses of Cd-exposed groups (Supplementary Fig. 1), except in female ApoE3 mice, where there was an apparent decrease in the alpha diversity in the low Cd exposure group (not statistically significant). Furthermore, the differences in alpha diversity were minimal in the basal conditions (i.e., no Cd) among all four sex-genotype groups (Supplementary Fig. 2). PCoA did not reveal changes in beta diversity between sexes or between different levels of Cd exposure. There was, however, a clear separation in beta diversity between the ApoE3-KI and ApoE4-KI genotypes (Fig. 2a–c), indicating that host genetics (ApoE3 vs. ApoE4) have a stronger influence on the gut microbiome configuration as compared to sex or chemical exposure.

### Compositional microbiome analysis

**Fig. 3:**
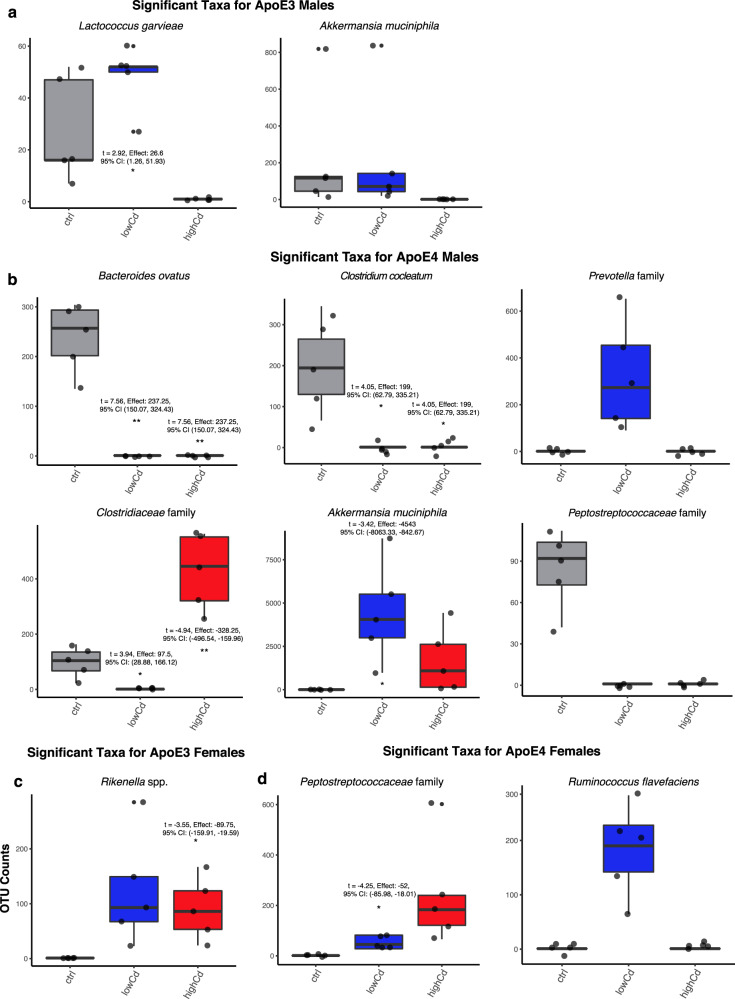
Significant microbiome changes by Cd exposure through analysis of composition of microbiomes (ANCOM). Bar plots depicting the difference in counts of significant classes between groups of different chemical exposure in **a** ApoE3 Males (*n* = 5 for all Cd groups), **b** ApoE4 Males (*n* = 5 for all Cd groups), **c** ApoE3 Females (*n* = 5 for all Cd groups), and **d** ApoE4 Females (*n* = 5 for all Cd groups). Significance was achieved if *p*-value < 0.05 from the ANCOM method. Asterisks indicate *p*-value < 0.05 from the post-hoc analysis when compared to the Control group (*t*-test: *p < 0.05, **p < 0.01). The *t*-statistic, effect size (difference in means compared to control) and 95% confidence intervals (CI) are given for all significant results from the *t*-tests.

We obtained relative abundance measures of the gut microbiome composition using QIIME2. There was an increase in the relative abundance of the class *Verrucomicrobiae* in ApoE4-KI male mice following exposure at the low Cd dose (Fig. 2d) which was due to an increase in *Akkermansia muciniphila* in this class (Fig. 3). An increase in *A. muciniphila* has been observed in AD patients in previous studies^28^. As shown in Supplementary Fig. 3, the male ApoE4 specific cadmium-mediated upregulation of *Akkermansia muciniphila* in large intestinal content was confirmed by qPCR (*N* = 5 per group). Consistent with the 16S rRNA sequencing data (Figs. 3 and 6b), the cadmium-mediated upregulation of *A. muciniphila* in male ApoE4 mice was more prominent by the low Cd exposure than the high Cd dose (Supplementary Fig. 3B). Also consistent with the 16S rDNA sequencing data (Figs. 3 and 6b), while the basal levels of *A. muciniphila* were minimal in the large intestinal content of all 4 groups, male ApoE3 mice had the highest basal level of *A. muciniphila* than the other 3 groups. We also noted a slight increase of *A. muciniphila* by the low Cd dose, but the RNA abundance remained low (Supplementary Fig. 3B). In addition, because 16S rDNA sequencing presents the relative abundance of each taxon in terms of percentage of OTUs, we further confirmed that the Cd-mediated upregulation of *A. muciniphila* in large intestinal content of male ApoE4 mice was not due to a systemic decrease in other bacteria by quantifying the gut microbiome using universal 16S rDNA degenerative PCR primers. We found no statistical differences in universal 16S rDNA abundance in the basal or Cd-exposed groups (Supplementary Fig. 3b).

ANCOM revealed that high Cd exposure reduced *Lactococcus garvieae* in the large intestinal content of male ApoE3-KI mice (Fig. 3a). Male ApoE4-KI mice had the greatest number of differentially regulated microbes by Cd exposure as compared to the other groups (Fig. 3b). Male ApoE4-KI mice were also the most susceptible group for Cd-induced AD-like neurotoxicity^5^. Specifically, both the low and high Cd doses markedly decreased *Bacteroides ovatus*, which is the predominant commensal intestinal microbe associated with an increase in IgG and IgA antibodies^29^. This aligns with a previous report that serum levels of both IgG and IgA are known to be lower in serum AD patients^30^. Both low and high doses of Cd exposure also decreased *Clostridium cocleatum*, which is a known beneficial commensal intestinal microbe that degrades mucin and protects against *Clostridium difficile* colonization in mice^31^. Low Cd dose increased *Prevotella* spp. (from 0 to approximately 300 OTU counts, asterisk not added due to zero values in control and high Cd dose groups), as well as *A. muciniphila*; both species are known microbial biomarkers in AD patients^28^. In addition, the relative abundance of the *Clostridiaceae* family was downregulated in the low Cd dose but was upregulated in the high Cd dose. The *Clostridiaceae* family, as well as the *Lactobacillaceae* family can produce short chain fatty acids (SCFAs) under anaerobic conditions^32^. Additionally, previous research has shown that the *Clostridiaceae* family was less abundant in AD patients.

Female mice in general had less differentially regulated taxa from Cd exposure (Fig. 3c and d). High doses of Cd increased the relative abundance of *Rikenella* spp. in the large intestinal content of female ApoE3-KI mice (low Cd dose also tended to increase this genus, although statistical significance was not reached) (Fig. 3c). Increased abundance of the *Rikenellaceae* family has been associated with AD patients; however, it is not clear whether *Rikenella* is the contributing genus in this family^10^.

In ApoE4-KI females, low Cd increased the relative abundance of the *Peptostreptococcaceae* family in the large intestinal content; the high Cd dose also tended to increase this microbe although statistical significance was not achieved (Fig. 3d). *Ruminococcus flavefaciens* was also increased in the low Cd group. *Peptostreptococcaecae* is a family of Gram-negative anaerobic bacteria with a fermentative type of metabolism. The *Peptostreptococcaecae* family was found to be less abundant in AD patients^10^. However, an increased abundance of the *Peptostrepococcaecae* family has also been found in patients with neurodevelopmental disorders^33^. Supplementation of *R. flavefaciens* has been shown to reduce the effects of depressive-like behavior. In regards to the gut-brain axis, there is evidence that *R. flavefaciens* affects gene networks in the brain^34^.

### Predicted functional annotation of the microbiome

PICRUSt2 was used to predict functional annotations of the microbiome for all four sex-genotype groups following Cd exposure. Among the four groups, ApoE3-KI males had the greatest number (51) of differentially regulated predicted pathways following Cd exposure. Interestingly, most of them were upregulated, except for the L-tryptophan biosynthesis pathway, which was downregulated by the high Cd dose (Supplementary Fig. 4). Most notably, carbohydrate metabolism, such as glycolysis, homolactic fermentation, and pathways related to the Krebs cycle were significantly increased in a dose–response manner following Cd exposure. Guanosine, adenosine, and pentose phosphate pathways, as well as pyrimidine and purine nucleotide synthesis pathways were all upregulated in ApoE3-KI mice, suggesting the role of Cd in accelerating DNA synthesis in microbiota. The biosynthesis of menaquinol and polyamines were also upregulated, and both of these molecules have been implicated in AD studies: menaquinol is the reduced form of menaquinone (vitamin K_2_); recent animal studies have suggested a positive association between vitamin K consumption and brain cells development and survival. Conversely, vitamin K antagonists may negatively influence cognitive domains like visual memory and fluency^35^. Polyamines are cations that can interact with DNA, RNA, and proteins. It has been shown that levels of polyamines decline with age and supplementation of this molecule can potentially increase life span^36^. Additionally, abnormal levels of polyamines have been associated with neurodegenerative processes that are commonly seen in AD^36^. Other predicted pathways that were up-regulated by Cd exposure included fermentation pathways such as homolactic fermentation, acetylene degradation, glycerol degradation, and lactose degradation, as well as the mevalonate pathway, which plays a critical role in eukaryotic metabolism.

ApoE4-KI males had fewer differentially regulated predicted pathways (10) following Cd exposure, and most of them were downregulated by Cd, except for the chitin derivatives degradation pathway, which was up-regulated by low dose of Cd (Supplementary Fig. 5). tRNA processing, amino acid (L-tryptophan, tyrosine, and phenylalanine) synthesis, fatty acid synthesis, anhydromuropeptides recycling, and coenzyme A (CoA) pathways were downregulated in the low Cd group. Four pathways, namely reductive acetyl coenzyme A pathway, L-tryptophan biosynthesis, isobutanoyl-CoA synthesis and gluconeogenesis, were downregulated in the high Cd group. Notably, reduced tryptophan input may lead to reduced trypotophan microbial metabolites, including indole-3-propionic acid (IPA).

There were no predicted pathways that were differentially regulated by Cd exposure in ApoE3-KI females, and there were 21 predicted functional pathways that were downregulated following Cd exposure in ApoE4-KI female mice (Supplementary Fig. 6). For example, the synthesis of lipid IVA, oleate, mycolate, palmitate, stearate, palmitoleate and (5Z)-dodecenoate and several other fatty acids was downregulated in a dose-dependent manner, suggesting that the ApoE4-cadmium gene-environment may lead to abnormal lipid metabolism. Unlike ApoE3-KI males, menaquinol synthesis (demethylmenaquinol-6) was decreased by Cd exposure in ApoE4-KI females. The degradation of inositol was significantly downregulated in the low Cd dose group. Inositol is a carbocylic sugar that is abundant in the brain. In a double-blind study, inositol was given to Alzheimer’s patients for one month. After the end of trial, language and orientation improved significantly in the inositol group with no serious side effects, suggesting that inositol supplementation may reduce neurodegenerative pathways caused by AD^37^.

### SCFA analysis

**Fig. 4:**
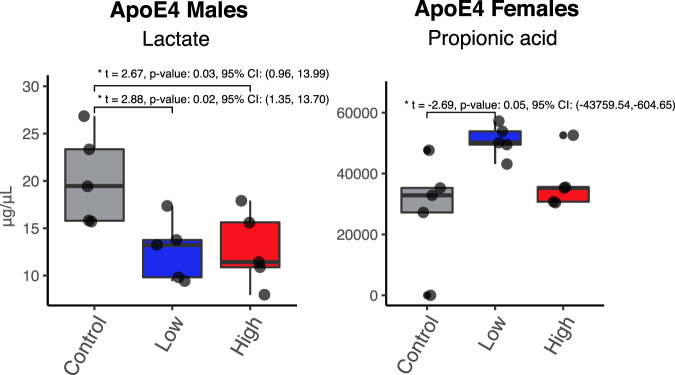
Levels of SCFAs are altered after cadmium exposure. Bar plots depicting the levels (μg/mL) of lactate in ApoE4-KI Males and propionic acid in ApoE4-KI females. Significance was determined through ANOVA for chemical groups compared to the Control group (*p*-value < 0.05). *n* = 5 for each sex and chemical group.

We quantified the levels of serum SCFA using GC-MS. Levels of lactate, a short chain hydroxy-fatty acid, were significantly decreased by both low and high Cd doses in ApoE4-KI males but not in mice of the other genotype or sex (Fig. 4 and Supplementary Figs. 7 and 8). Although lactate is not classified as a SCFA, it is a substrate of several common SCFAs in the intestinal lumen. In a previous study using an AD mouse model (APP/PS1 mice), there was an association between decreased lactate content and the presence of amyloid beta plaques as well as reduced amounts of neurons and oligodendrocytes^38^. Furthermore, the role of lactate has shifted from being a waste metabolite to an essential molecule for neuronal function and long-term memory. The astrocyte-neuron lactate shuttle model states that astrocytes metabolize glucose to lactate, which neurons use as an energy source^39^. Therefore, a reduction in circulating lactate may reduce CNS lactate supply, leading to energy deprivation, and neuronal damage. In addition, propionic acid was up-regulated in serum of ApoE4-KI females by the low Cd dose (Fig. 4). This SCFA has been shown to interfere with Aβ1-40 oligomerization^40^. There was an increase in propionic acid in a study assessing the use of a novel formulation of lactic acid bacteria and *bifidobacteria* in a triple-transgenic mouse model of AD, suggesting that an increase of propionic acid may be a compensatory mechanism against the progression of AD^41^.

### Liver transcriptome

**Fig. 5:**
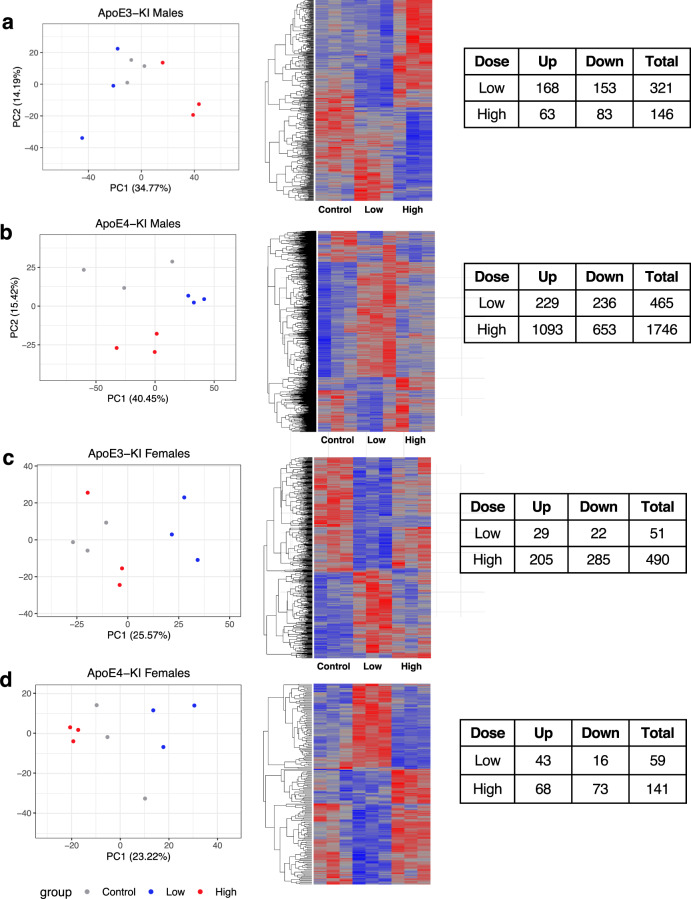
PCA plots, heatmaps, and table of significantly regulated genes. Principal component analysis (PCA) plots were used to represent the differences in transcriptomes between different Cd levels in ApoE3-KI males (**a**), ApoE4-KI males (**b**), ApoE3-KI females (**c**) and ApoE4-KI females (**d**). Heatmaps show the hepatic gene expression levels; significance was determined through pairwise comparisons with FDR < 0.05. *n* = 3 for each sex and chemical group.

We quantified the liver transcriptome using RNA-Seq. In ApoE3-KI Males, a total of 321 genes and 146 genes were significantly differentiated in the low and high Cd dose group respectively. These genes were clustered in two primary groups: genes that were upregulated in the high dose and genes that were downregulated in the high dose when compared to the other two treatment groups (Fig. 5a). Compared to ApoE3-KI males, there were substantially more genes significantly dysregulated in ApoE4-KI males; a total of 465 genes and 1746 genes were significantly upregulated in the low and high dose group, respectively. Two main clusters of genes were formed in this group; the dendrogram split into a group of genes that were upregulated and a group of genes that were downregulated in the low dose group (Fig. 5b). In ApoE3-KI females, a total of 51 genes and a total of 490 genes were significantly regulated in the low and high dose group respectively (Fig. 5c). Even fewer genes were disrupted in ApoE4-KI females. A total of 59 genes and a total of 141 genes were significantly differentiated in the low and high dose groups (Fig. 5a–d). In the principal components analysis, there was greater separation in the principal components of the dose group in the ApoE4-KI genotypes, suggesting that the Cd causes greater disruption in the global liver transcriptome in the ApoE4 genotype (Fig. 5a–d).

**Fig. 6:**
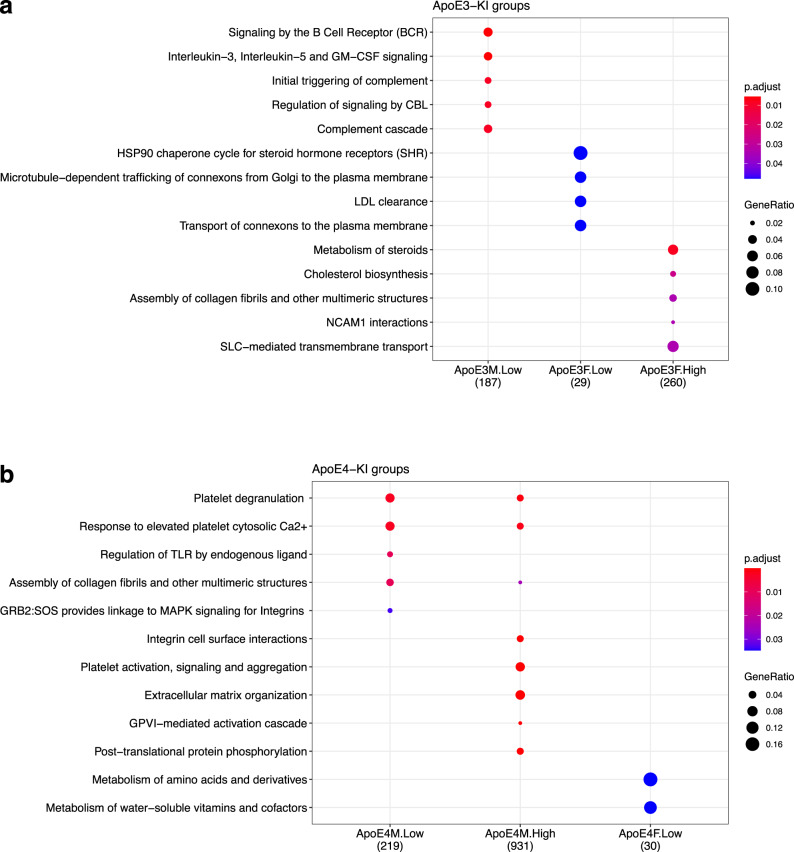
Significant disrupted pathways in the liver. Dot plots of significantly disrupted pathways in ApoE3-KI groups (**a**) and ApoE4-KI groups (**b**) from ReactomePA. Significance is determined by *p* < 0.05. The gene ratio is defined as ratio of genes found in the specific pathway to all the significantly differentiated genes.

From the global transcriptome analysis, we used ReactomePA to determine if there were any pathways that were significantly disrupted by genotype and Cd exposure. In the ApoE3-KI groups, low doses of Cd in males led to disruptions in the innate and adaptive immune response such as signaling by the B Cell Receptor, initial triggering of the complement and interleukin-3, interleukin-5 and GM-CSF signaling. In ApoE3-KI females, low Cd exposure led to disruptions in microtubule-dependent trafficking of connexons from Golgi to the plasma membrane, suggesting that Cd exposure affects protein coding, production, and trafficking of membrane protein. Cd exposure profoundly affected pathways related to lipid metabolism in ApoE3-KI females. In low doses, Cd disrupted the HSP90 chaperone cycle for steroid hormone receptors and LDL clearance, whereas in high doses, Cd disrupted steroid metabolism such as cholesterol biosynthesis (Fig. 6a).

There were several overlapping pathways in both the low and high Cd exposure groups in ApoE4-KI males. Platelet degranulation, response to elevated platelet cytosolic Ca^2 +^, and assembly of collagen fibrils and other multimeric structures were significantly regulated by Cd, suggesting its role in fibrosis. Integrin, cell surface interactions, platelet activation, signaling and aggregation, extracellular matrix organization, CPVI-mediated activated cascade (which involves the platelet immune receptor downstream of platelet activation)^42^, and post-translational protein phosphorylation were significantly disrupted in the high Cd group of ApoE4-KI males. To note, platelet activation has been shown to be an important mechanism in initiating pro-inflammatory responses during liver damage and liver diseases^43–47^. Upon further examination of transcriptome, there was a marked increase in the expression of platelet activation genes in the ApoE4-KI male groups exposed to a low dose of Cd (Supplementary Fig. 9). In the livers of the low Cd dose exposed ApoE4-KI females, only two pathways, metabolism of amino acid and derivatives and metabolism of water-soluble vitamins and cofactors, were disrupted by Cd exposure. No pathways were significantly regulated in liver of the high Cd dose-exposed ApoE4-KI females (Fig. 6).

**Fig. 7:**
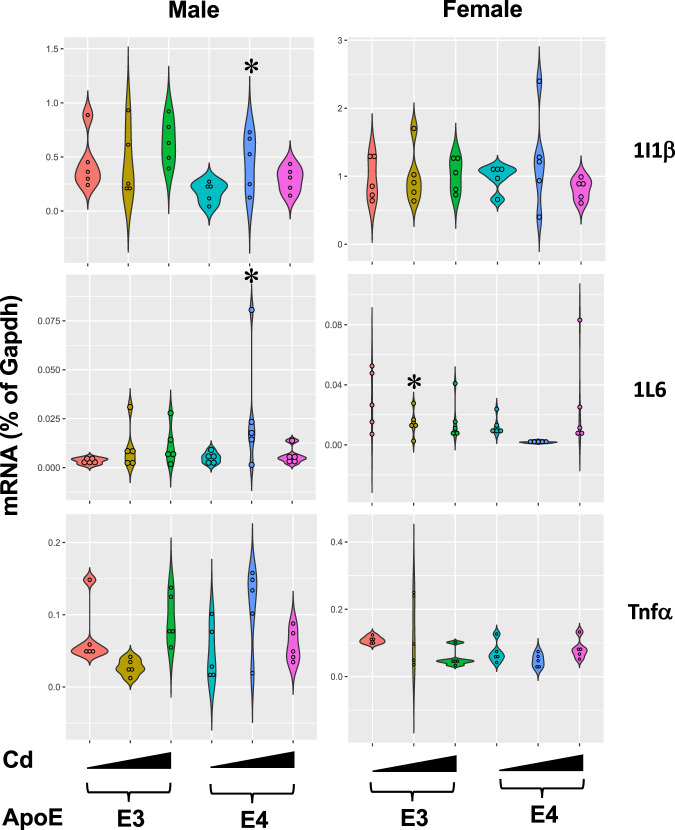
RT-qPCR results of inflammation genes. The mRNA expression of selected liver genes involved in inflammation (interleukin 1β [IL-1β], as well as its target genes tumor necrosis factor α [TNFα] and IL-6) was quantified in livers of vehicle- or Cd-exposed male and female mice of either ApoE3 or ApoE4 genotypes (*n* = 5/sex/genotype/exposure). *n* = 5 for each sex and chemical group. Asterisks indicate *p* < 0.05 when compared to the control.

We verified the increased expression of inflammation-related genes using RT-qPCR (Fig. 7) since there was an increase in the expression of inflammation genes according to our RNA-seq analysis. As shown in Fig. 7, IL-1β mRNA was up-regulated by Cd low dose uniquely in livers of male ApoE4-KI but not in livers of male ApoE3-KI mice or female mice of either genotypes. Cd high dose had minimal effect on IL-1β gene expression. Corresponding to the male ApoE4-specfiic increase in IL-1β mRNA, the IL-1β-target gene TNFα was also up-regulated in livers of male ApoE4-KI mice. TNFα mRNA was downregulated by Cd low dose in livers of female ApoE3-KI mice. Another known target gene of IL-1β, namely IL-6, tended to be up-regulated in livers of male ApoE4-KI mice, although statistical significance was not achieved. In summary, both the mRNA of the pro-inflammatory cytokine IL-1β as well as its target genes appear to be uniquely increased by low doses of Cd in the livers of male ApoE4-KI mice, suggesting a functional elevation of pro-inflammatory signaling pathways.

Because hepatic xenobiotic biotransformation is critically involved in Alzheimer’s disease, we also quantified important host drug-metabolizing enzyme expression in liver using RT-qPCR (Supplementary Fig. 10)^13,14^. These genes were selected based on the differential analysis from the liver RNA-Seq datasets. Interestingly, male ApoE4-KI mice were the only group that was not responsive to Cd-mediated alterations in the mRNAs of the drug-metabolizing P450 enzymes in liver. Except for the Cd-mediated upregulation in Cyp1a2 mRNA in livers of female ApoE4-KI mice, all of the other differentially regulated P450s were downregulated by Cd exposure, including a decrease in Cyp2a5 mRNA by a low dose of Cd in livers of male ApoE3-KI mice and by the Cd high dose in livers of the female ApoE4 mice; a general trend of decrease in a Cd dose–response manner in Cyp2b9 mRNA in livers of male ApoE3-KI, female ApoE3-KI, and female ApoE4-KI mice; as well as a decrease in Cyp2c38, Cyp2d9, and Cyp2d26 mRNAs by the high Cd dose in livers of male ApoE3-KI mice. Because the drug-metabolizing P450s are involved in bioactivation of potentially harmful metabolites, we speculate that the lack of hepatic response to Cd-mediated down-regulation P450s in male ApoE4-KI mice may result in an accumulation of more harmful metabolites, although additional metabolomic studies are needed to validate this speculation.

Due to the role of the liver in xenobiotic metabolism, we hypothesize that liver toxicity may lead to systemic toxicity. Since differentially regulated genes lead to liver toxicity, reversal of these genes through certain drugs can mitigate Cd toxicity. For example, for genes that were significantly up-regulated, chemicals that down-regulate these genes could act as potential therapeutics. Using the results from the global transcriptome analysis, we employed a hypothesis generating approach for finding remedies for Cd toxicity using the LINCS L1000 database. The majority of potential therapeutics from this analysis were known anti-cancer treatments. In ApoE3-KI males, mitoxantrone was predicted to reverse up-regulated genes whereas selumetinib and radicicol was predicted to reverse downregulated genes in low dose groups. In ApoE4-KI males, withaferin-a and radicicol were hypothesized to reverse up-regulated genes and ropinirole HCl was hypothesized to reverse downregulated genes caused by a low dose Cd exposure. In ApoE4-KI males exposed to a high dose of Cd, pelitinib, vorinostat, and afatinib were predicted to combat Cd toxicity caused by significantly up-regulated genes. Dasantib was hypothesized to mitigate Cd toxicity in ApoE3-KI females exposed to a low dose of Cd. Alvocidib, dinaciclib, cleastrol, mocetinostat, and pracinostat were predicted to mitigate effects from a high dose of Cd exposure in the same sex and genotype. In ApoE4-KI females, gefitinib and geldanamycin were predicted to reverse the effects of significantly regulated genes cause by Cd exposure (Supplementary Table 3).

### Pearson’s correlation analysis between gut microbiome and host liver genes involved in inflammation and xenobiotic biotransformation

**Fig. 8:**
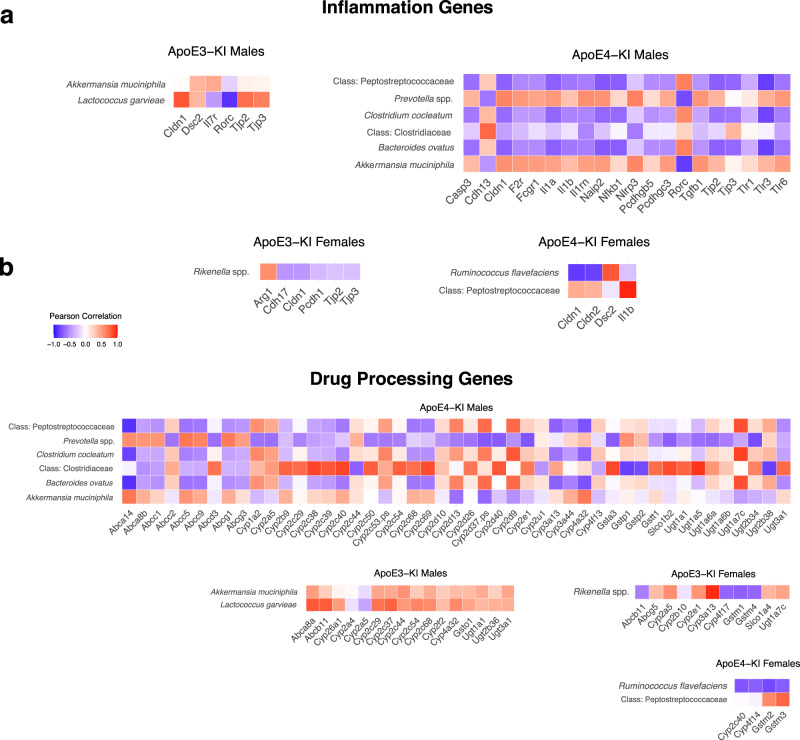
Pearson correlation plots of significant genes involved in inflammation and drug metabolism. Pearson correlation plots of significant taxa to inflammation genes (**a**) and drug processing genes (**b**) in all genotype-sex groups.

Using the Pearson correlation measure, we characterized the relationship between significantly regulated taxa to significantly regulated inflammation and drug processing genes (DPGs). We defined a correlated gene-taxa pair if the Pearson correlation measure was greater than 0.7. Regarding inflammation-related pathways, ApoE4-KI males had the most numbers of correlated bacteria-host inflammation-related gene pairs. Most notably, *Prevotella* spp. and *A. muciniphila* were positively associated with most of the pro-inflammation-related genes in liver. To note, increased abundance *Prevotella* spp. has been observed in multiple chronic inflammatory diseases in humans^48^, whereas *A. muciniphila* is anti-inflammatory^49^, but is also known to be enriched in AD patients^28^, suggesting a compensatory mechanism in response to pro-inflammatory cascade. Several inflammation genes were also found negatively correlated with significant taxa in ApoE4-KI males. Tight junction protein 2 (*Tjp2*), tight junction protein 3 (*Tjp3*), neuronal apoptosis inhibitory protein 2 (*Naip2*), nuclear factor kappa B subunit 1 (*Nfkb1*), and toll-like receptor 3 (*Tlr3*) were all negatively correlated with *Bacteroides ovatus*; *Naip2, Nfkb1*, *Tlr3*, and *Tjp2* were negatively correlated with the *Peptostreptococcaeceae* family. Lastly, *Tlr3* was negatively correlated with *C. cocleatum*. In ApoE3-KI males, Claudin 1 (*Cldn1*) and tight junction protein (*Tpr1*) were positively correlated with *Lactococcus garvieae;* RAR related orphan receptor C (*Rorc*) was negatively correlated with *L. garvieae*. No inflammation genes were strongly correlated with the corresponding significant taxa in ApoE3-KI females. In ApoE4-KI females, *Cldn1* and claudin 2 (*Cldn2*) were negatively correlated with *R. flavefaciens* while desmocollin 2 (*Dsc2*) was positively correlated with *R. flavefaciens* (Fig. 8a).

With regards to DPGs, ApoE4-KI males also had the most numbers of correlated bacteria-host DPG pairs. The *Clostridiaceae* family was positively associated with the most Cyp2 family members, multiple phase-II enzymes (Gsta3, Gstt1, several Ugt family members), as well as the Slco1b2 hepatic xenobiotic uptake transporter. In *B. ovatus*, UDP glucuronosyltransferase 1 (*Ugt1a7c*), cytochrome P450, family 2, subfamily d, polypeptide 13 (*Cyp2d13*), cytochrome P450, family 2, subfamily d, polypeptide 37, pseudogene (Cyp2d37-ps) were positively correlated; cytochrome P450, family 2, subfamily a, polypeptide 32 (*Cyp2a32*), cytochrome P450, family 3, subfamily a, polypeptide 13 (*Cyp3a13*), and ATP binding cassette subfamily A member 14 (*Abca14*) were negatively correlated. *Ugt1a7c* and *Cyp2d37-ps* were positively correlated with the *Peptostreptococcaceae* family; *Abca14* was negatively correlated with this taxon. ATP binding cassette subfamily A member 8b (*Abca8b*) was positively associated with *Prevotella* spp. *Abca14* was negatively associated with *C. cocleatum*. ATP binding cassette subfamily B member 11 (*Abcb11*) was positively correlated with *L. garvieae* in ApoE3-KI males. No genes were strongly correlated in ApoE3-KI females. Only one DPG (Gstm2) was strongly positively associated with a significant species (*R. flavefaciens*) in ApoE4-KI females (Fig. 8b).

## Discussion

**Fig. 9:**
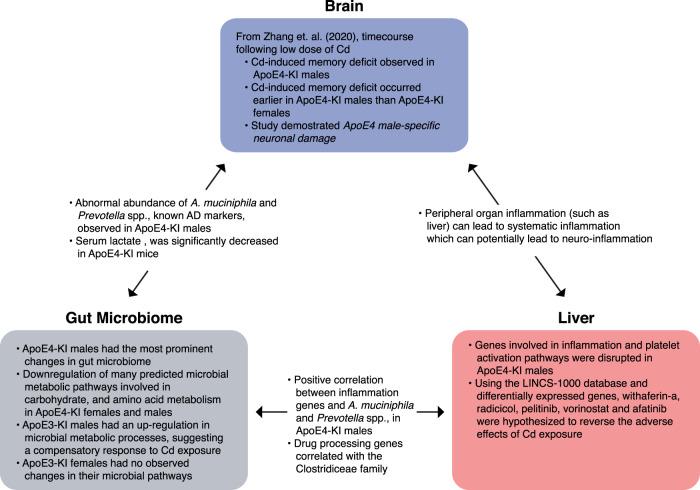
Overall summary. Summary and working hypothesis regarding how gut-liver-brain axis may contribute to the differences in the susceptibility to Cd-induced neurotoxicity^5^ between genotypes (ApoE3 vs. ApoE4) and sexes.

A previous study demonstrated that oral Cd exposure in drinking water increased the susceptibility of ApoE4-KI male mice to learning and memory deficits^5^. Using the same cohort of mice, the present study serves to investigate the association between the brain-gut-liver axis and the sex- and genotype-specific susceptibility to Cd-induced neurotoxicity. We found that the most susceptible group to Cd-induced neurotoxicity, namely ApoE4-KI males, also had the most prominent changes in gut microbiome signatures involved in inflammation and energy deprivation, as well as host hepatic genes involved in inflammation and xenobiotic biotransformation. The dysregulation of these pathways within the gut-liver axis may provide additional mechanisms of the overall Cd-mediated toxicity in the CNS and other organs (Fig. 9).

ApoE4-KI males had the most prominent changes in their gut microbiota, including an increase in well-established human AD hallmarks (*A. muciniphila* and *Prevotella* spp.)^11^, a reduction in the IgG/IgA-producing *B. ovatus* (consistent with the decrease in serum IgG/IgA observed in AD patients)^30^, and an increase in several pro-inflammatory taxa (*Clostridiaceae* family and *Prevotella* spp).

Although, *A. muciniphila* is thought to be a beneficial microbe in patients with diabetes and obesity, its benefits for AD populations may not be clear. In our study, an increase in the abundance of *A. muciniphila* did not correlate with an increase in SCFAs, such as acetic acid, propionic acid and succinate. In fact, we observed a decrease (not significant) in succinate in ApoE3-KI male mice. In fact, there is growing literature that suggests the relationship between *A. muciniphila* and CNS health may be more complex than once thought. *A. muciniphila* overabundance has been observed in patients with Parkinson’s disease, autism spectrum disorder and multiple sclerosis. It is hypothesized that an increase in this microbe may lead to the increased inflammation that are characteristic of these neurological disorders^50^. Furthermore, the regulation of *A. muciniphila* in AD or AD models appears to be species-specific. In the APPPS1 mouse model, which is another genetic model that recapitulates AD in mice, this microbe is decreased. These observations suggest the caution that needs to be made while interpreting results between AD in humans and AD mouse models and suggests that ApoE4-KI mice may serve as a more accurate model in recapitulating Cd-induced neurodegenerative diseases in humans.

Based on our gut microbiome results, the common human ApoE3 gene may have a protective effect against Cd exposure. Using PICRUSt2, a predictive annotation tool for microbiome data, the altered microbiota composition was predicted to exhibit the down-regulation of many essential microbial pathways involved in nutrient and energy homeostasis in ApoE4-KI mice (Supplementary Figs. 3, 4), suggesting decreased energy and protein production in response to Cd exposure and failure to preserve energy in the genetically susceptible mouse strain.. Conversely, a 2015 study showed that Cd exposure in mice increased key pathways involved in energy metabolism in the livers of wild type mice^27^. Our study showed that mice that carry the common human ApoE3 allele also had a predicted increase in key pathways involved in energy metabolism in the gut microbiome. Together, these two studies align with each other and suggest a compensatory mechanism to combat against Cd-induced toxicity within the gut-liver axis.

Dysbiosis of the liver transcriptome was most severe in ApoE4-KI males. Specifically, pathways involved in platelet activation were disrupted in ApoE4-KI males. Genes in these pathways were largely up-regulated by low dose of Cd, which aligns with our observation of enriched *A. muciniphila* in the low Cd group of ApoE4-KI males. There is growing evidence that platelets play an important role in liver homeostasis. As key players in thrombosis and hemostasis, it has been suggested that platelets directly fuel tumor growth in the liver. In a cohort of patients with biopsy proven hepatocellular carcinoma (HCC), tumor size and platelet count were positively correlated, suggesting an association between the development of HCC and platelet activation^51^. Furthermore, the administration of anti-platelet drugs such as aspirin has shown to reduce the severity of liver injury and fibrosis in animal models of chronic liver disease^52^. Adding to the neurotoxic toxic effects of Cd exposure, these results point to the potential hepatotoxic role of Cd exposure through the enrichment of platelet activation pathways in ApoE4-KI males.

Using the LINCS-1000 database, we uncovered distinct chemicals that may reverse the adverse effects of Cd exposure based on hepatic expression data. Within the ApoE4-KI male group, anti-cancer drugs such as withaferin-a, radicicol, pelitinib, vorinostat, and afatinib were hypothesized to mitigate the effects of Cd toxicity. Specifically, withaferin-a has been shown to protect against liver injury through the induction of Nrf2-dependent cytoprotective enzyme expression^53^. Ropinirole HCl, typically used for Parkinson’s disease (PD)^54^, was also predicted as a potential treatment for Cd toxicity in an AD-susceptible population, however, studies also show that there is an association between the onset of dementia and usage of this chemical^55^.

Consistent with our previous microbiome results (Fig. 3), there were fewer distinct chemicals (gefitinib and geldanamycin) for reversing the adverse effects of Cd exposure in ApoE4-KI females. Originally prescribed as an anti-cancer therapy, gefitinib has been shown to be a potential treatment of Aβ-induced memory loss by antagonizing the epidermal growth factor receptor^56^. Geldanamycin, a Hsp90 inhibitor, has shown to be successful at ameliorating tau and Aβ burden. However, other Hsp90 inhibitors have been recommended instead due to high hepatotoxicity and off-target effects of geldanamycin^57^.

Although, we observed significant changes in the gut microbiota and hepatic transcriptome separately, characterizing the crosstalk between the gut, brain, and liver is vitally important for understanding Cd-induced AD. Short-chain fatty acids (SCFAs) are the main metabolites produced by bacterial fermentation in the gut microbiota and are hypothesized to play an important role in the gut-brain axis (PMID: 31123355)^58^. Serum lactate was significantly decreased in ApoE4-KI mice. Although we cannot directly determine if this was caused by dysbiosis of the microbiota, previous studies have shown that the *Bacteroides* family are producers of succinate, lactate, acetate, and propionate. Our study observed a significant decrease in this microbe, which could explain the decrease in lactate and succinate in ApoE4-KI male mice. The *Bacteroides* family is highly abundant and accounts for 20% of the human colon microbiota; a significant decrease in this microbiome could lead to fundamental changes in the function of the gut microbiota^59^. Further in vitro culture studies, such as quantifying SCFA levels in Cd-exposed *Bacteroides ovatus* culture, are needed to strengthen this association. Although butyric acid has been implicated in a number of neurodegenerative diseases (PMID: 29095058), there was no significant associations between levels of butyric acid, genotype, and Cd. This suggests that while Cd correlates with AD, it does not constitute the entire mechanism of this neurodegenerative disease.

Alterations to the host liver transcriptome likely contribute to the genotype-specific susceptibility to Cd partly through the liver’s communication with the gut microbiome. First, we showed that ApoE4-KI males had more differentially regulated liver genes involved in inflammation (positively correlated with *Prevotella* spp. and *A. muciniphila*) as well genes involved in xenobiotic biotransformation (positively associated with the *Clostridiceae* family). The other genotypes and sexes had much fewer differentially regulated host liver genes, and although Cd low dose-exposed ApoE3-KI males had predicted enrichment in inflammation-related pathways, it was minimally correlated with the microbiome, indicating that the direct influence of Cd on the host liver is independent from the gut microbiome. An increase in neuroinflammation was recognized as a central mechanism in AD; the peripheral organs (including liver) may contribute to systemic inflammation and the subsequent neuroinflammation^60^.

Neuroinflammation is a well-recognized important contributing factor for neurodegenerative diseases including AD^61^. Although we did not quantify neuroinflammation or systemic inflammation in this study, a number of inflammation genes were significantly correlated with *A. muciniphila* and *Prevotella* spp. in the hepatic transcriptomes of ApoE4-KI males. Our study, using RT-qPCR, demonstrated a significant increase in IL-1β and TNFα, two pro-inflammatory cytokines, in ApoE4-KI male mice exposed to the low Cd dose. IL-1β, a pro-inflammatory cytokine, together with IL-6 and TNF activate local immune cells and attract other leukocytes to the liver, leading to a chronic inflammatory state^62,63^. Increased hepatic inflammation has also been observed in another study where wild-type mice were exposed to Cd in drinking water^27^, therefore our study aligns with evidence from previous literature. There have been studies showing that liver inflammation may have detrimental effects on the brain^64^, whereas systemic inflammation may lead to neuroinflammation^65^. Further studies on proinflammatory cytokine levels in serum and in brain are needed to confirm the importance of liver-derived inflammation on Cd-induced neurotoxicity.

There were observed sex differences between ApoE4-KI males and females. In males, the intestinal microbiota likely contributed to the ApoE genotype-specific susceptibility to Cd-induced neurotoxicity. Although the exact mechanism is unknown, the microbiome may be more susceptible to sex-specific differences. In a 2019 study, the gut microbiomes of 57 men and women were characterized. The results of the study demonstrated that sex steroids may be associated with different microbiome compositions. Although speculative, these differences could lead to a more stable microbiome in females^66^. Furthermore, we observed greater dysbiosis of the transcriptome in ApoE4-KI males. There are several studies that have demonstrated evidence of sex differences in the expression of DPGs and inflammation genes^67–69^. For example, a 2011 study showed that the release of growth hormone was associated with the difference in the expression of sulfotransferases between sexes^70^. There are a variety of different factors that can lead to differing gene expression between sexes; further research is needed in order to elucidate the mechanisms that lead to different transcriptomes between sexes following Cd exposure.

One of the key findings in this study was the observed genotype and gender specific gut dysbiosis caused by Cd exposure. It has been shown in previous literature that alterations in the gut microbiomes occur before the development of key pathological features of AD. In a time-course study, APP/PS1 mice had decreased gut diversity and distinct changes in inflammation-related bacteria, such as *Akkermansia*, before exhibiting amyloidosis and plaque-localized neuroinflammation^71^. Furthermore, an increase in systemic inflammation, potentially caused by our observed increase in pro-inflammatory species and expression of host inflammation genes, can induce neuroinflammation and lead to tau phosphorylation^72^. Peripheral inflammation can lead to the activation of glial cells via the production of both pro- and anti-inflammatory cytokines. Persistent activation of microglial cells, especially in aged rats, can lead to the progression of neurodegenerative diseases such as AD^73^. Taken together, these results demonstrate how gut dysbiosis and subsequent increase in systemic inflammation can contribute to the molecular hallmarks of AD.

There are several limitations in the present study: first, we examined the terminal time point of Cd exposure whereas Zhang et. al. followed up Cd-induced neurotoxicity over a time course^5^; it would be informative to monitor the time course of the microbiome in this mouse model in order to determine the precise window of time for microbial changes relative to the disease phenotype. In addition, although we found correlations between microbe levels and SCFA levels, it is unclear if the serum SCFAs were derived from the host or the microbiome; exposing germ-free mice with Cd may provide a more causal relationship in future studies.

In summary, this study is among the first multi-omic investigation to provide evidence that the heavy Cd produces compositional and functional changes in the gut microbiome, as well as dysregulation of host liver transcriptome in carriers that are genetically susceptible to Alzheimer’s disease. Although being observational in nature, our study has established the groundwork for future mechanistic studies. Within the context of host genetic risk factor × gut microbiome × environmental exposures, our study is the first to unveil the phenomenon that gut dysbiosis following exposure to cadmium at human tissue burden relevant levels is uniquely regulated by the both the host ApoE genotype and sex, with distinct microbial biomarkers identified in the male mice carrying the human ApoE4 gene allele, which is the strongest known genetic risk factor for Alzhemier’s disease. Considering it has been shown in the NHANES study that cadmium is an environmental chemical that is associated with significantly increased risk of Alzheimer’s disease^74^, as well as the recent Congress report (February 2021) and the Baby Food Safety Act 2021 both pointing out the significant food contamination of cadmium in US, our findings on the ApoE genotype and sex specific gut microbial response following cadmium exposure is both important and timely.

## Methods

### Animals

Humanized ApoE3 and ApoE4 knock-in (ApoE3-KI and ApoE4-KI) mice were obtained from Dr. Nobuyo Maeda from the University of North Carolina at Chapel Hill and have been characterized before^4^. Mice were housed in standard conditions (12 h light/dark cycle) at the University of Washington animal facilities. All mice were housed according to the Association for Assessment and Accreditation of Laboratory Animal Care (AAALAC) International guidelines, and studies were approved by the Institutional Animal Care and Use Committee (IACUC) at the University of Washington.

### Chemicals

Acetic acid was purchased from Thermo Fisher Scientific (Fair Lawn, NJ). Propionic acid, isobutyric acid, butyric acid, 2-methylbutyric acid, isovaleric acid, valeric acid, 2-methylpentanoic acid, 3-methylpentanoic acid, isocaproic acid, caproic acid, 2-methylhexanoic acid, 4-methylhexanoic acid, heptanoic acid, hexanoic acid-6,6,6-d_3_ internal standard, N-tert-Butyldimethylsilyl-N-methyltrifluoroacetamide (MTBSTFA), and methoxyamine hydrochloride were purchased from Sigma-Aldrich (St. Louis, MO).

### Cd exposure

At 28 days, humanized ApoE3 knock-in (ApoE3-KI) and ApoE4 knock-in (ApoE4-K1) mice were weaned and randomly separated into groups of 3-5 mice per cage^5^. At 8 weeks of age, drinking water with 0.6 mg/L CdCl_2_ was introduced to the low Cd treatment group and drinking water with 3 mg/L CdCl_2_ was introduced to the high Cd treatment group. The CdCl_2_ water was prepared with a stock solution (Cat. 202908, MilliporeSigma, St. Louis, Missouri) and replaced every week. In order to characterize the persistent effects of Cd, mice in the treatment group were exposed for 14 weeks and then switched back to normal drinking water. Behavior experiments were completed as reported before^5^, and samples of the same cohort were collected when mice reached 75-weeks of age (Fig. 1). There were 4 mice in each genotype, exposure, and sex category. This sample size was calculated based on previously published studies and a statistical power analysis using Snedecor and Cochran’s sample size formula *n* = 1 + 2 C(s/*d*)^2^; *d* is the expected magnitude of difference of the outcome between groups, s is the standard deviation of the variable of interest. C is a constant based off Type I error and power; C is 10.51 for 5% Type I Error of and 90% power^75^.

### 16S rDNA sequencing

Using a similar method described previously^76–78^, total DNA was isolated from the large intestinal content of male and female ApoE3-KI and ApoE4-KI mice (*n* = 4–5). Briefly, samples were prepared using an E.Z.N.A. DNA Stool Kit (Omega Bio-tek Inc., Norcross, GA) per the manufacturer’s instructions. The concentration of DNA was quantified using a Qubit 2.0 Fluorometer (Life Technologies, Grand Island, NY). The integrity and quality of DNA samples were confirmed using an Agilent 2100 Bioanalyzer (Agilent Technologies Inc., Santa Clara, CA). The V4 hypervariable region of 16S rDNA was amplified and sequenced using a HiSeq 2500 second generation sequencing platform (250-bp paired-end) (Novogene Corporation Inc., Sacramento, CA). The 16S rRNA sequencing data is available on Dryad (https://datadryad.org/stash/share/1OuvcAutrdilVacoYc6B4CUhHTQg7ZKtlie6hDzSi3U).

### qPCR validation of microbial DNA

Selected differentially regulated bacteria were validated by quantitative polymerase chain reaction (qPCR) using Bio-Rad CFX384 Real-Time PCR Detection System (Hercules, CA). The 16S rRNA primers for the detection of *Akkermansia muciniphila* were designed on the basis of the 16S rRNA sequences of these bacteria and these primer sequences were reported in our previous publications^76,79^ and in Supplementary Table 4. The primers recognizing the universal bacterial 16S rRNA sequences were provided by the National Gnotobiotic Rodent Resource Center core facilities, University of North Carolina. All primers were synthesized by Integrated DNA Technologies (Coralville, IA). The abundances of the genomic DNA were expressed as mean delta-delta cycle value (ddCq) of the quantitative PCR as normalized per 5 ng DNA template (Supplementary Table 4).

### RNA-Seq of liver transcriptome

Total RNA was isolated from livers of Cd or vehicle exposed ApoE3-KI and ApoE4-KI mice (*n* = 4 per exposure per sex) using RNA zol Bee reagent (Tel-Test Inc., Friendswood, TX). The RNA concentration was determined using a NanoDrop 1000 Spectrophotometer (Thermo Fisher Scientific, Waltham, MA) at 260 nm. The quality of RNA was evaluated by formaldehyde-agarose gel electrophoresis by visualizing the 28S and 18S rRNA bands under UV light. The RNA integrity value (RIN) was examined using an Agilent 2100 Bioanalyzer (Agilent Technologies Inc.), and samples with a RIN value above 8.0 were used for RNA-Seq. cDNA libraries were prepared using a Clontech cDNA library prep kit (Clontech Laboratories Inc., Mountain View CA), and were sequenced using a NextSeq 500 sequencing platform (75 bp paired end). The RNA-Seq data is available on Dryad (https://datadryad.org/stash/share/1OuvcAutrdilVacoYc6B4CUhHTQg7ZKtlie6hDzSi3U).

### Reverse transcription quantitative polymerase chain reaction (RT-qPCR) of liver genes involved in inflammation

The mRNA expression of selected liver genes involved in inflammation (interleukin 1β [IL-1β], as well as its target genes tumor necrosis factor α [TNFα] and IL-6) was quantified in livers of vehicle- or Cd-exposed male and female mice of either ApoE3 or ApoE4 genotypes (n = 5/sex/genotype/exposure). Total RNA was reverse-transcribed into cDNA using a High Capacity cDNA Reverse Transcription Kit (Life Technologies, California). The resulting cDNA products were diluted 1:10 and were amplified by qPCR, using the Sso Advanced Universal SYBR Green Supermix in a Bio-Rad CFX384 Real-Time PCR Detection System (Bio-Rad, Hercules, California). The primers for all qPCR reactions were synthesized by Integrity DNA Technologies (Coralville, Iowa). Primer sequences are shown in Supplementary Table 3. Data are expressed as % of the expression of the housekeeping gene glyceraldehyde 3-phosphate dehydrogenase (Gapdh, Supplementary Table 4). Data were analyzed using two-way analysis of variance (ANOVA) followed by Tukey’s post hoc test (*p* < 0.05). Asterisks represent statistically significant difference as compared to the appropriate vehicle controls.

### Short chain fatty acid (SCFA) quantification using gas chromatography mass spectrometry (GC-MS)

Using similar methods from a 2019 publication of a metabolome analysis^80^, frozen plasma samples were first thawed overnight at 4 °C. Then, 20 μL of each sample was mixed with 20 μL hexanoic acid-6,6,6-d_3_ (internal standard), 30 μL sodium hydroxide solution (NaOH, 0.1 M in water) and 430 μL methanol (MeOH). The pH of the mixture was 9. Following storage at −20 °C for 20 min and centrifugation at 14,000 rpm for 10 min, 450 μL of supernatant were collected and sample pH was adjusted to 10 by adding 30 μL of NaOH:H2O [1:4]. Samples were then dried, reconstituted in 40 μL of methoxyamine hydrochloride in pyridine (20 mg/mL), and stored at 60 °C for 90 min. Afterward, 60 μL of N-methyl-N-tert-butyldimethylsilyltrifluoroacetamide were added and stored at 60 °C for 30 min. Each sample was then vortexed for 30 s and centrifuged at 14,000 rpm for 10 min. Finally, 70 μL of supernatant were collected from each sample for GC-MS analysis.

GC-MS experiments were performed on an Agilent 7820A GC-5977B MSD system (Santa Clara, CA) by injecting 1 µL of prepared samples. Helium was used as the carrier gas with a constant flow rate of 1.2 mL/min. The separation of metabolites was achieved using an Agilent HP-5ms capillary column (30 m × 250 µm × 0.25 µm). The column temperature was maintained at 60 °C for 1 min, and then increased at a rate of 10 °C/min to 325 °C and held at this temperature for 10 min. Mass spectral signals were recorded after a 4.9 min solvent delay.

### Statistical analysis

Microbiome data was analyzed using QIIME 2 (version 2.1) using DADA2 for denoising^81^. For the diversity analyses, the Shannon index was used to quantify alpha diversity and the Bray-Curtis dissimilarity measure was used to quantify beta diversity. Using this measure, principle coordinates analysis (PCoA) was used to visualize changes in the microbiome composition between groups. Because microbiome data is inherently compositional, analysis of composition of microbiomes (ANCOM) was used to detect significant differences in microbiome taxa between the control group, low, and high Cd exposure groups^82^. The two-sided *t*-test was used in the post-hoc analysis in order to determine which pair (i.e., control vs. low or control vs. high) was significantly different. Certain pairwise comparisons could not be made since there were zero counts across all samples for a particular species in a genotype-sex group. For example, if there are zero counts for *Prevotella* spp. in the high dose group in ApoE4-KI females, then we cannot compute a *t*-statistic for the control vs. high comparison. Functional profiles of microbial communities was predicted using PICRUSt2 (Phylogenetic Investigation of Communities by Reconstruction of Unobserved States version 2)^83^. After correcting for multiple testing using Benjamini–Hochberg, ANOVA was used to determine significant pathways altered by Cd exposure.

For liver transcriptome data, FASTQ files with paired-end sequence reads were mapped to the mouse genome (UCSC mm10) using HISAT (Hierarchical Indexing for Spliced Alignment of Transcripts)^84^. The resulting SAM (sequence alignment/map) files were converted to its binary form (BAM: binary alignment/map) and sorted using SAMtools (verison 1.2)^85^. Transcript abundances were estimated with featureCounts (part of the Subread package, version 1.5.3)^86^ using the UCSC mm10 as the reference annotation. RUVSeq (version 1.18.0) was used to estimate factors of unwanted variation from the residuals of the count data. EdgeR (version 3.26.8) was used with the RUVSeq output in the differential expression analysis. A gene was considered significantly differentiated if the false discovery rate (FDR) < 0.05. Principal components analysis (PCA) plots and heatmaps (R package gplots) were generated to visually display the transcriptome changes caused by genotype and cadmium exposure. ReactomePA (R package: v1.28.0) was used to determine which pathways were significantly regulated (adjusted *p*-value < 0.05) in each genotype and sex group.

The list of differentially regulated genes for each genotype and sex group was split into upregulated genes (log fold count > 0) and downregulated genes (log fold count < 0). Using the Library of Integrated Network Based Cellular Signatures (LINCS) L1000 database, Enrichr (https://amp.pharm.mssm.edu/Enrichr/) was used to determine potential chemicals that could negate the effects of cadmium exposure for each genotype and sex group. Pearson correlation plots were generated in order to investigate whether compositional changes were correlated with significantly regulated drug processing genes or inflammatory genes.

In the SCFA analysis, a Student’s *t*-test was used to determine whether SCFA levels were significantly disrupted by Cd within the same sex and genotype. Measurements for the microbiome analysis, liver transcriptome analysis, and SCFA analysis were all taken from distinct samples.

### Reporting summary

Further information on research design is available in the Nature Research Reporting Summary linked to this article.

## Supplementary information

Supplementary Information

Description of Additional Supplementary Files

Supplementary Data 1

Reporting Summary

## Data Availability

The 16S rRNA sequencing data is available on Dryad (https://datadryad.org/stash/share/1OuvcAutrdilVacoYc6B4CUhHTQg7ZKtlie6hDzSi3U). The RNA-Seq data is available on Dryad (https://datadryad.org/stash/share/1OuvcAutrdilVacoYc6B4CUhHTQg7ZKtlie6hDzSi3U). Short and medium chain fatty data can be found in Supplementary Data 1.
