# Supplementary material for: Cadmium exposure modulates the gut-liver axis in an Alzheimer’s disease mouse model
Source: Commun Biol. 2021 Dec 15;4:1398. doi: 10.1038/s42003-021-02898-1 (PMC8674298; doi:10.1038/s42003-021-02898-1)
Supplement: Supplementary file 1 — Supplementary Information [file 42003_2021_2898_MOESM1_ESM.pdf]

CADMIUM EXPOSURE PERSISTENTLY MODULATES THE GUT-LIVER AXIS IN AN ALZHEIMER'S DISEASE MOUSE MODEL

Angela Zhang[1], Megumi Matsushita[1], Liang Zhang[1], Hao Wang[1], Xiaojian Shi[2], Haiwei Gu[2], Zhengui Xia[1], and Julia Yue Cui[1]

Supplementary Figure 1: Alpha diversity plots separated by sex and genotype; Shannon index, a commonly-used and accurate measure of alpha diversity, of (**a**) ApoE3-KI Females; (**b**) ApoE4-KI Females; (**c**) ApoE3-KI Males; and (**d**) ApoE4-KI Males. Data were analyzed using QIIME 2 as described in MATERIALS AND METHODS. Error bars represent the standard error within sex and genotype.

Supplementary Figure 2: Alpha diversity (Shannon index) separated by sex and genotype of mice at basal levels (no Cd exposure. Data were analyzed using QIIME 2 as described in MATERIALS AND METHODS. Error bars represent the standard error within sex and genotype.

Supplementary Figure 3: qPCR universal bacteria (a) and *A. muciniphila* (b) in large intestinal content of male and female ApoE3 and ApoE4 mice following exposure to vehicle, the low Cd dose (0.6 mg/L), and the high Cd dose (3mg/L). Data are expressed as ddCq per 5 ng DNA. Asterisks: $p < 0.05$ (Analysis of variance).

Supplementary Figure 4: Predicted functional annotation for ApoE3-KI Males; PiCRUST2 was used to generate the predicted functional annotation profiles. The heatmap depicts predicted pathway values for ApoE3-KI males; red indicates an enrichment of the pathway and blue indicates decreased pathway activity. ANOVA was used to determine significant pathways. Asterisks indicate FDR < 0.05.

Supplementary Figure 5: Predicted functional annotation for ApoE4-KI Males; PiCRUST2 was used to generate the predicted functional annotation profiles. The heatmap depicts predicted pathway values for ApoE4-KI males; red indicates an enrichment of the pathway and

blue indicates decreased pathway activity. ANOVA was used to determine significant pathways. Asterisks indicate FDR < 0.05.

Supplementary Figure 6: Predicted functional annotation for ApoE4 Females; PiCRUST2 was used to generate the predicted functional annotation profiles. The heatmap depicts predicted pathway values for ApoE4-KI females; red indicates an enrichment of the pathway and blue indicates decreased pathway activity. ANOVA was used to determine significant pathways. Asterisks indicate FDR < 0.05. Note: there were no differentially regulated pathways in ApoE3-KI females.

Supplementary Figure 7: Levels of SCFAs following cadmium exposure: box plots depicting various levels of SCFAs in ApoE3-KI males and ApoE4-KI males. Asterisks mark significance at $p < 0.05$ (t-test).

Supplementary Figure 8: Levels of SCFAs following cadmium exposure: box plots depicting various levels of SCFAs in ApoE3-KI females and ApoE4-KI females. Asterisks mark significance at $p < 0.05$ (t-test).

Supplementary Figure 9: Heatmap of genes involved in platelet activation pathways in ApoE4-KI males following exposure to no Cd, a low dose of Cd, and a high dose of Cd.

Supplementary Figure 10: RT-qPCR of selected drug-metabolizing P450s in livers of male and female ApoE3 and ApoE4 mice following exposure to vehicle, the low Cd dose (0.6 mg/L), and the high Cd dose (3mg/L). Data are expressed as % of the house keeping gene Gapdh. Asterisks: $p < 0.05$ (Analysis of variance).

Supplementary Table 1: 16S rDNA Sequencing number of reads

Supplementary Table 2: Liver Transcriptome Mapping Statistics

Supplementary Table 3: Comparison of the cadmium effect on the liver transcriptome and the LINCS database.

Supplementary Table 4: PCR primer sequences for inflammation genes, universal bacteria, *A. muciniphila* and CYP genes.

Supplementary Figure 1: Alpha diversity plots separated by sex and genotype; Shannon index, a commonly-used and accurate measure of alpha diversity, of (a) ApoE3-KI Females; (b) ApoE4-KI Females; (c) ApoE3-KI Males; and (d) ApoE4-KI Males. Data were analyzed using QIIME 2 as described in MATERIALS AND METHODS. Error bars represent the standard error within sex and genotype.

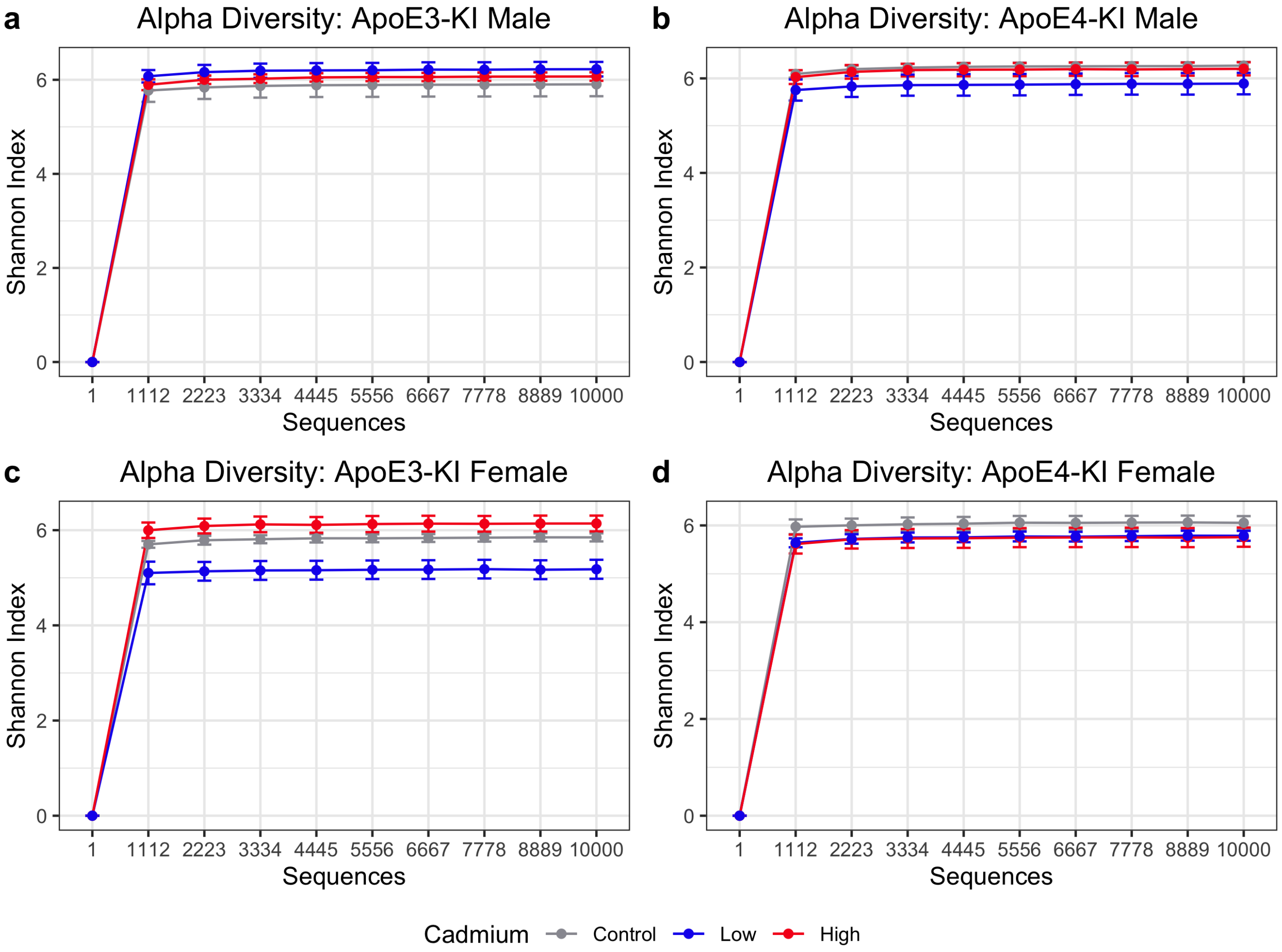

Supplementary Figure 2. Alpha diversity (Shannon index) separated by sex and genotype of mice at basal levels (no Cd exposure. Data were analyzed using QIIME 2 as described in MATERIALS AND METHODS. Error bars represent the standard error within sex and genotype.

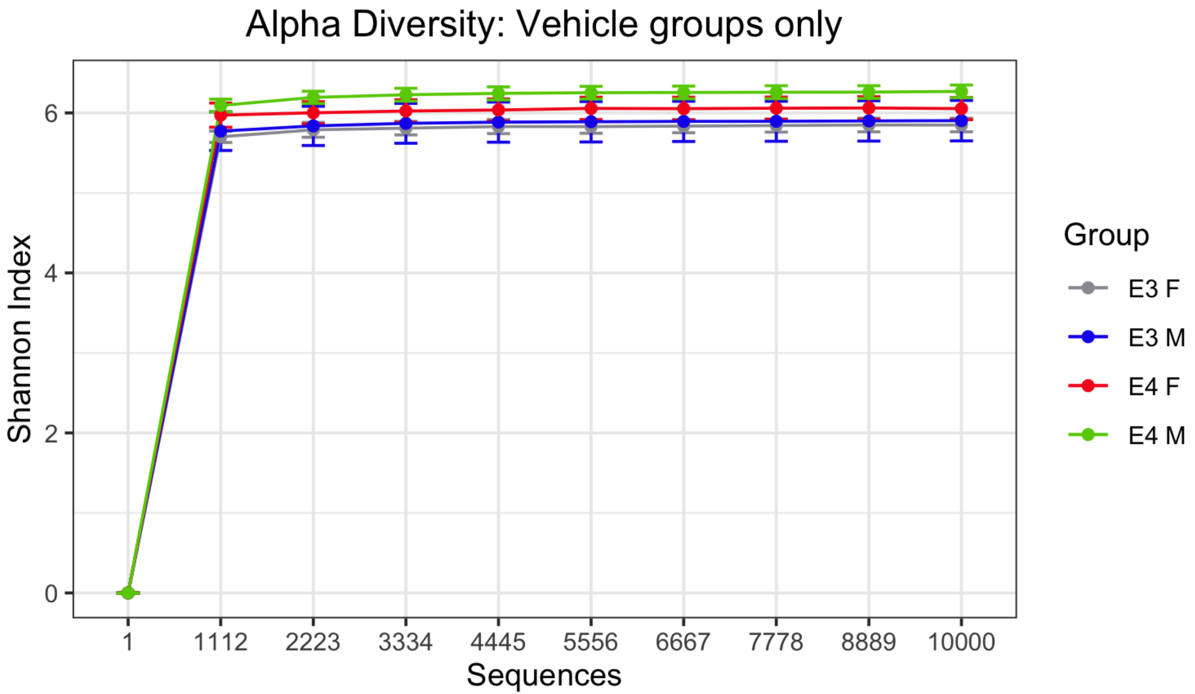

Supplementary Figure 3: qPCR universal bacteria (a) and A. muciniphila (b) in large intestinal content of male and female ApoE3 and ApoE4 mice following exposure to vehicle, the low Cd dose (0.6 mg/L), and the high Cd dose (3mg/L). Data are expressed as ddCq per 5 ng DNA. Asterisks: $p < 0.05$ (Analysis of variance).

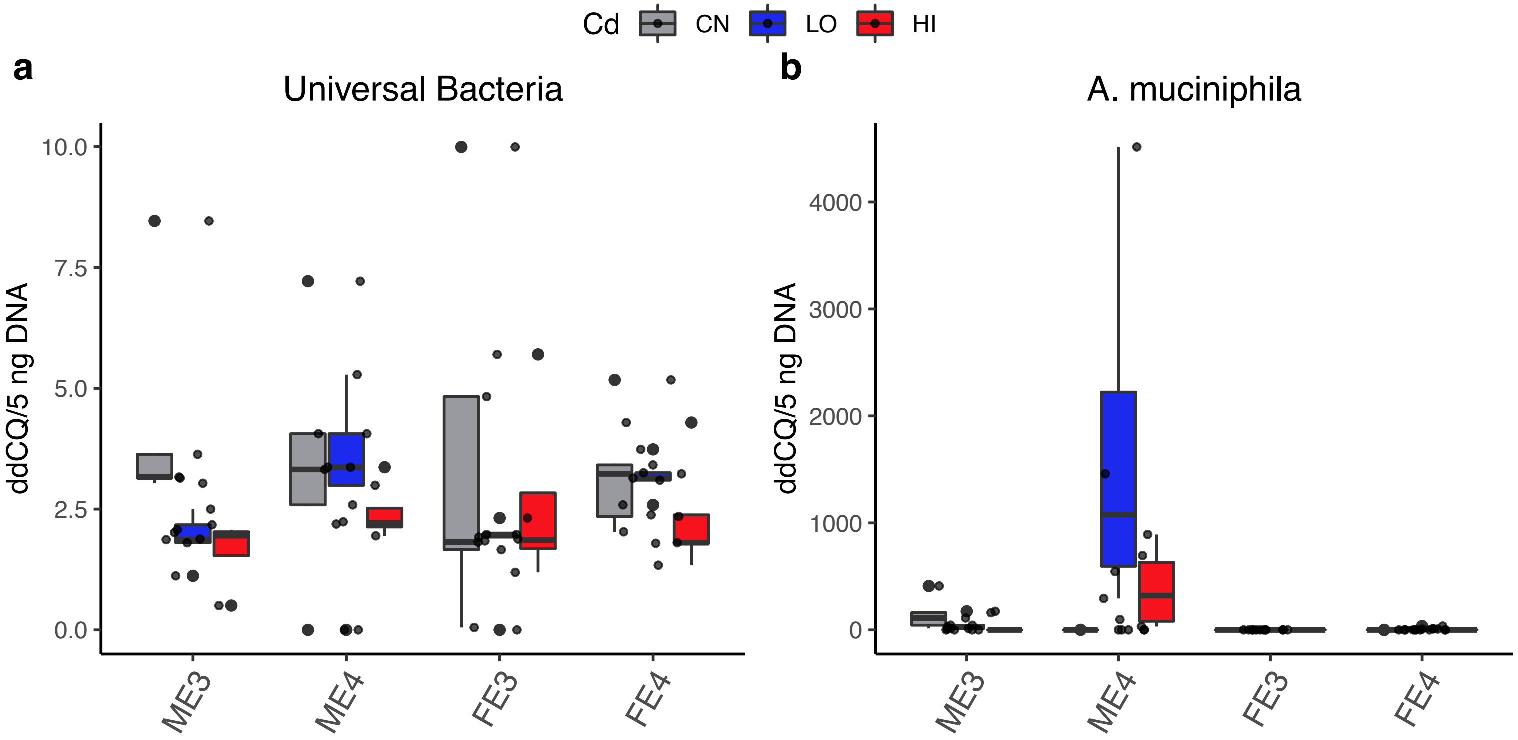

Supplementary Figure 4: Predicted functional annotation for ApoE3-KI Males; PiCRUST2 was used to generate the predicted functional annotation profiles. The heatmap depicts predicted pathway values for ApoE3-KI males; red indicates an enrichment of the pathway and blue indicates decreased pathway activity. ANOVA was used to determine significant pathways. Asterisks indicate FDR < 0.05.

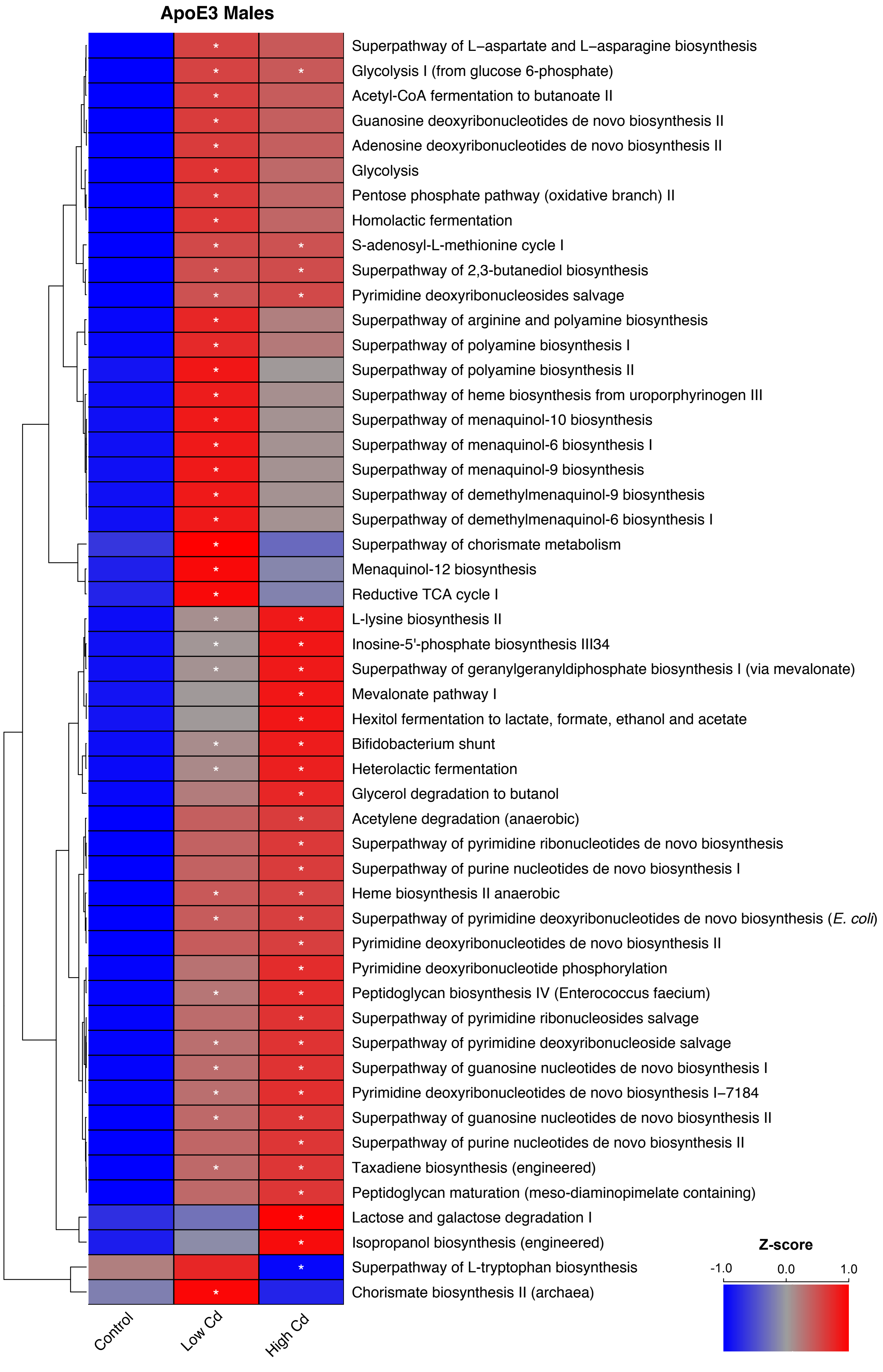

Supplementary Figure 5: Predicted functional annotation for ApoE4-KI Males; PiCRUST2 was used to generate the predicted functional annotation profiles. The heatmap depicts predicted pathway values for ApoE4-KI males; red indicates an enrichment of the pathway and blue indicates decreased pathway activity. ANOVA was used to determine significant pathways. Asterisks indicate FDR < 0.05.

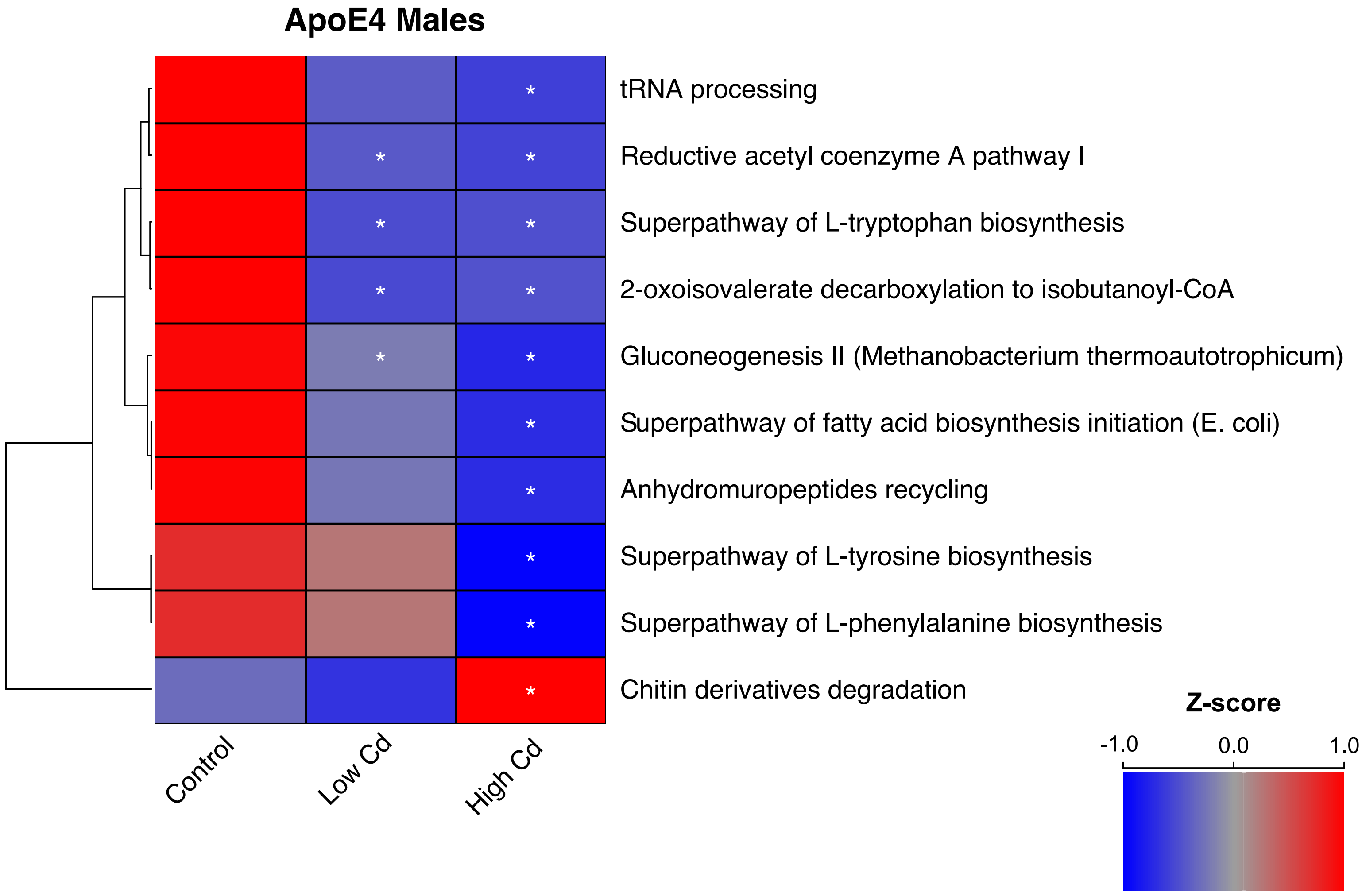

Supplementary Figure 6: Predicted functional annotation for ApoE4 Females; PiCRUST2 was used to generate the predicted functional annotation profiles. The heatmap depicts predicted pathway values for ApoE4-KI females; red indicates an enrichment of the pathway and blue indicates decreased pathway activity. ANOVA was used to determine significant pathways. Asterisks indicate FDR < 0.05. Note: there were no differentially regulated pathways in ApoE3-KI females.

ApoE3 Females
(none)

ApoE4 Females

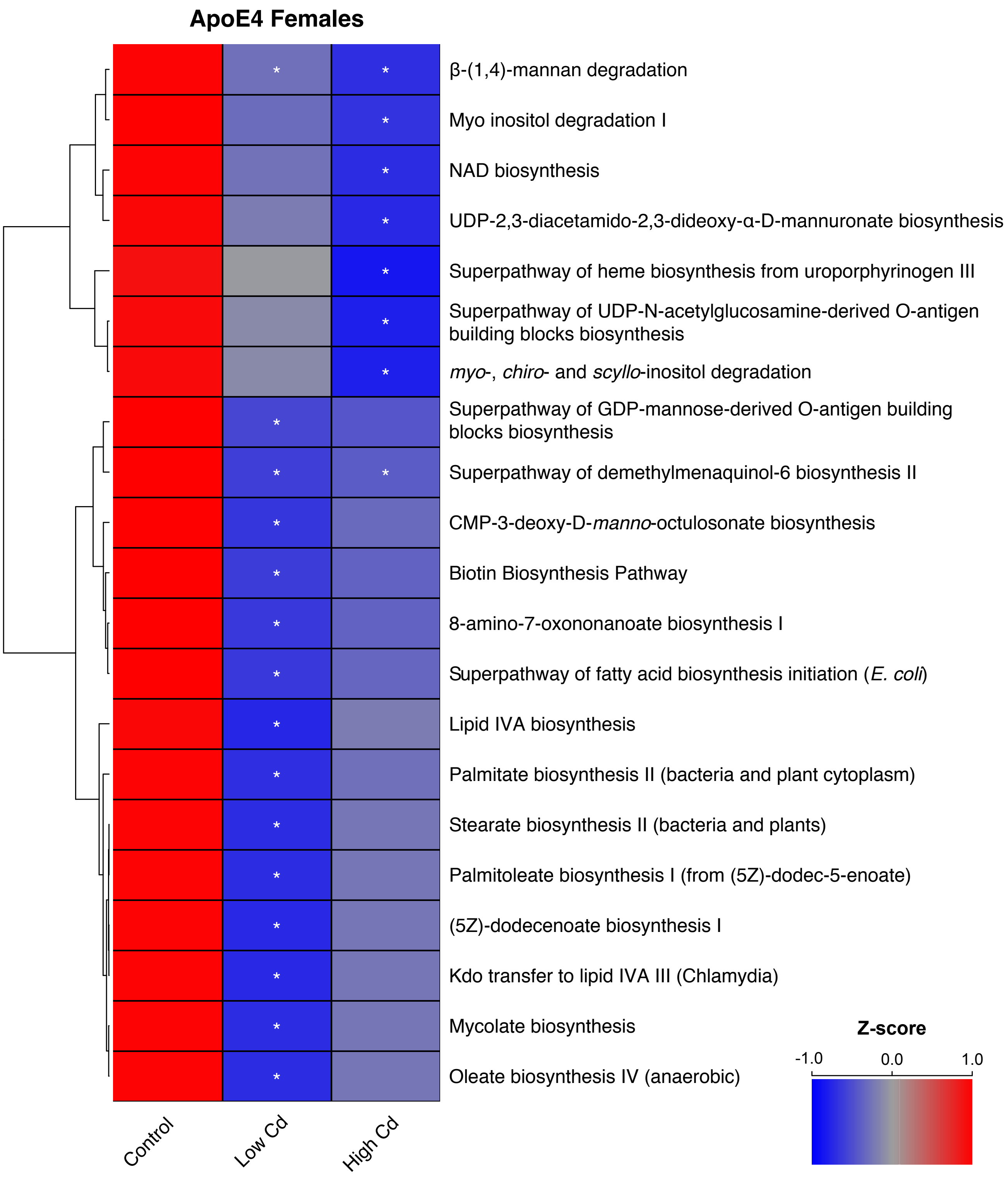

Supplementary Figure 7: Levels of SCFAs following cadmium exposure: box plots depicting various levels of SCFAs in ApoE3-KI males and ApoE4-KI males. Asterisks mark significance at $p < 0.05$ (t-test).

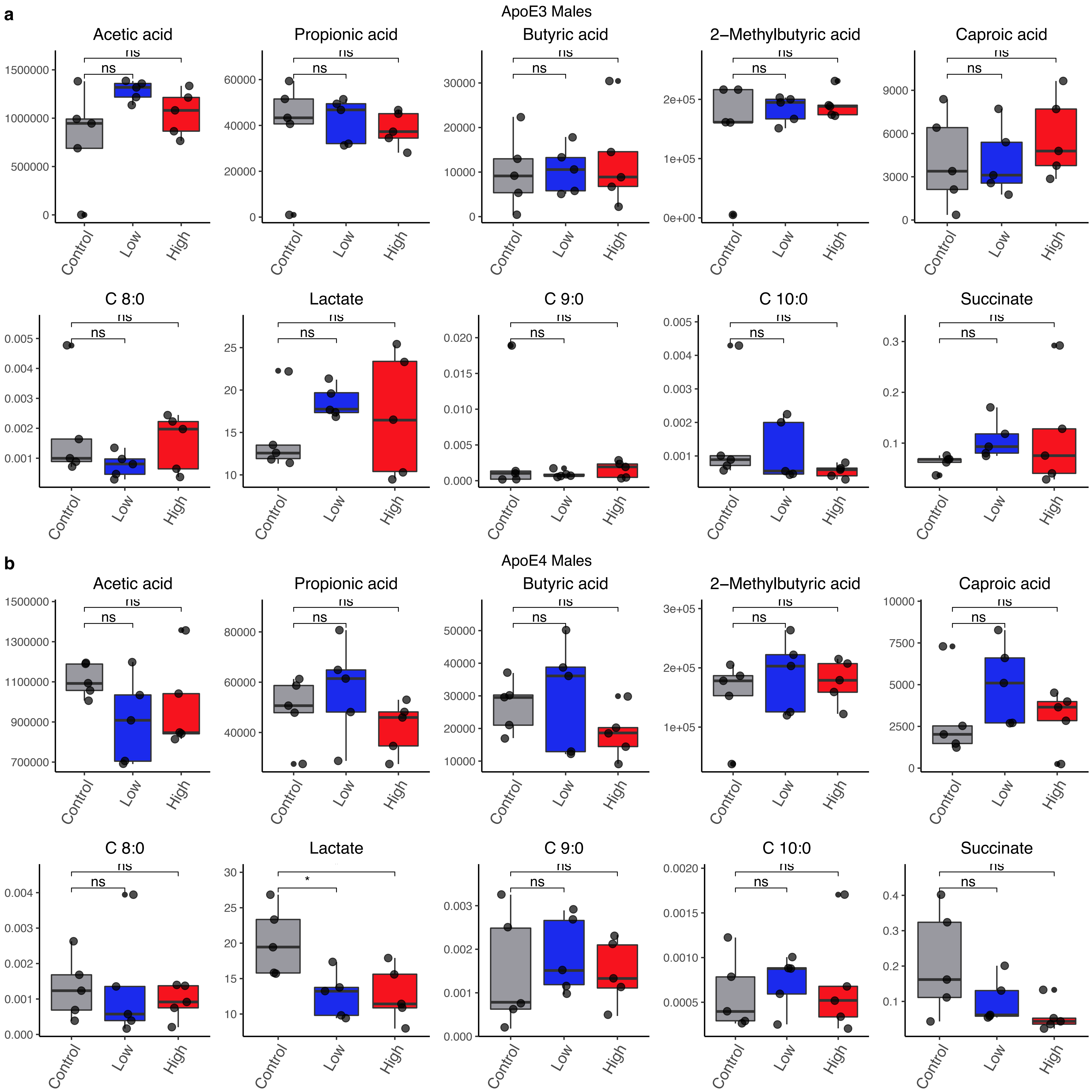

Supplementary Figure 8: Levels of SCFAs following cadmium exposure: box plots depicting various levels of SCFAs in ApoE3-KI females and ApoE4-KI females. Asterisks mark significance at $p < 0.05$ (t-test).

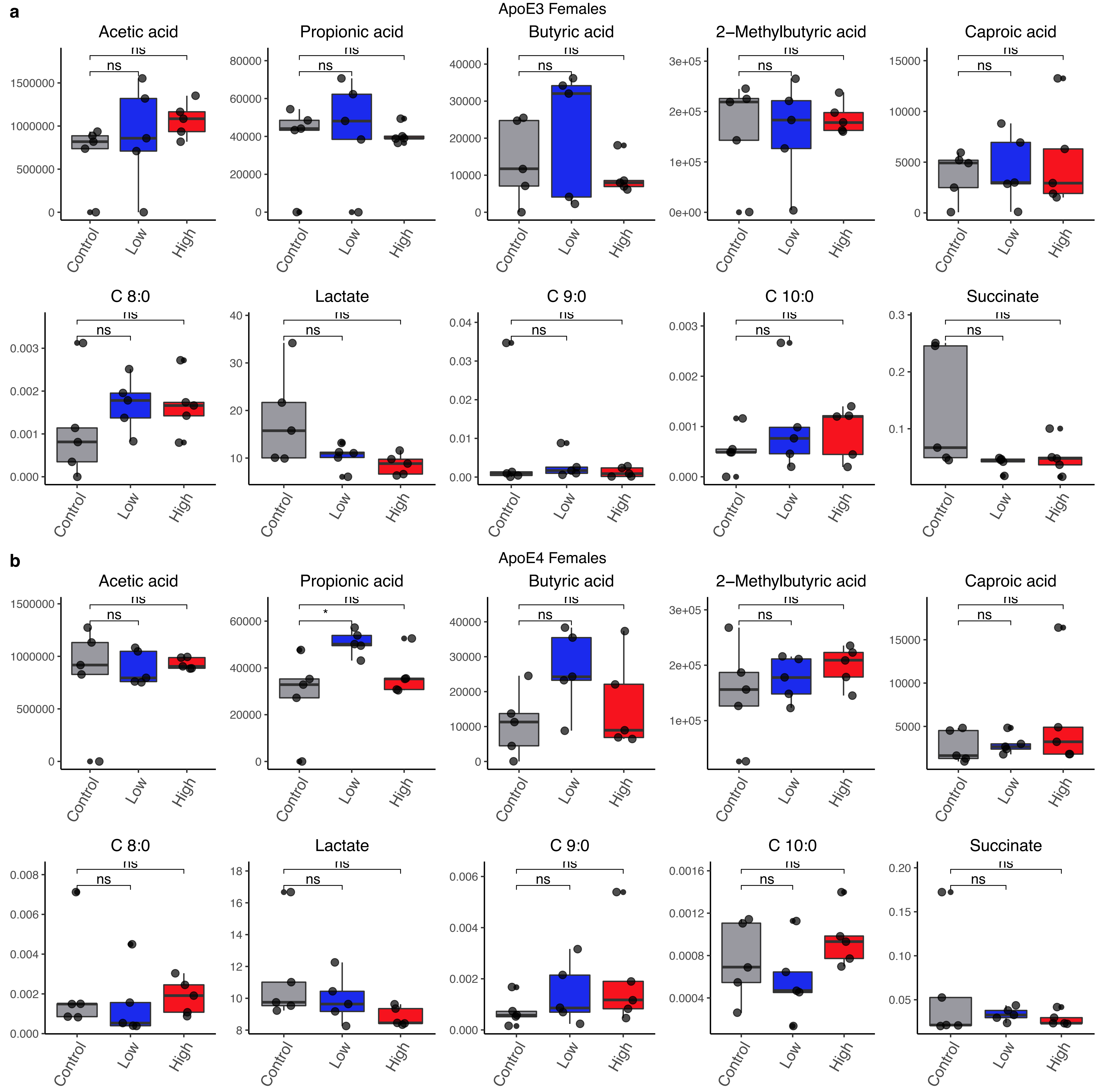

Supplementary Figure 9: Heatmap of genes involved in platelet activation pathways in ApoE4-KI males following exposure to no Cd, a low dose of Cd, and a high dose of Cd.

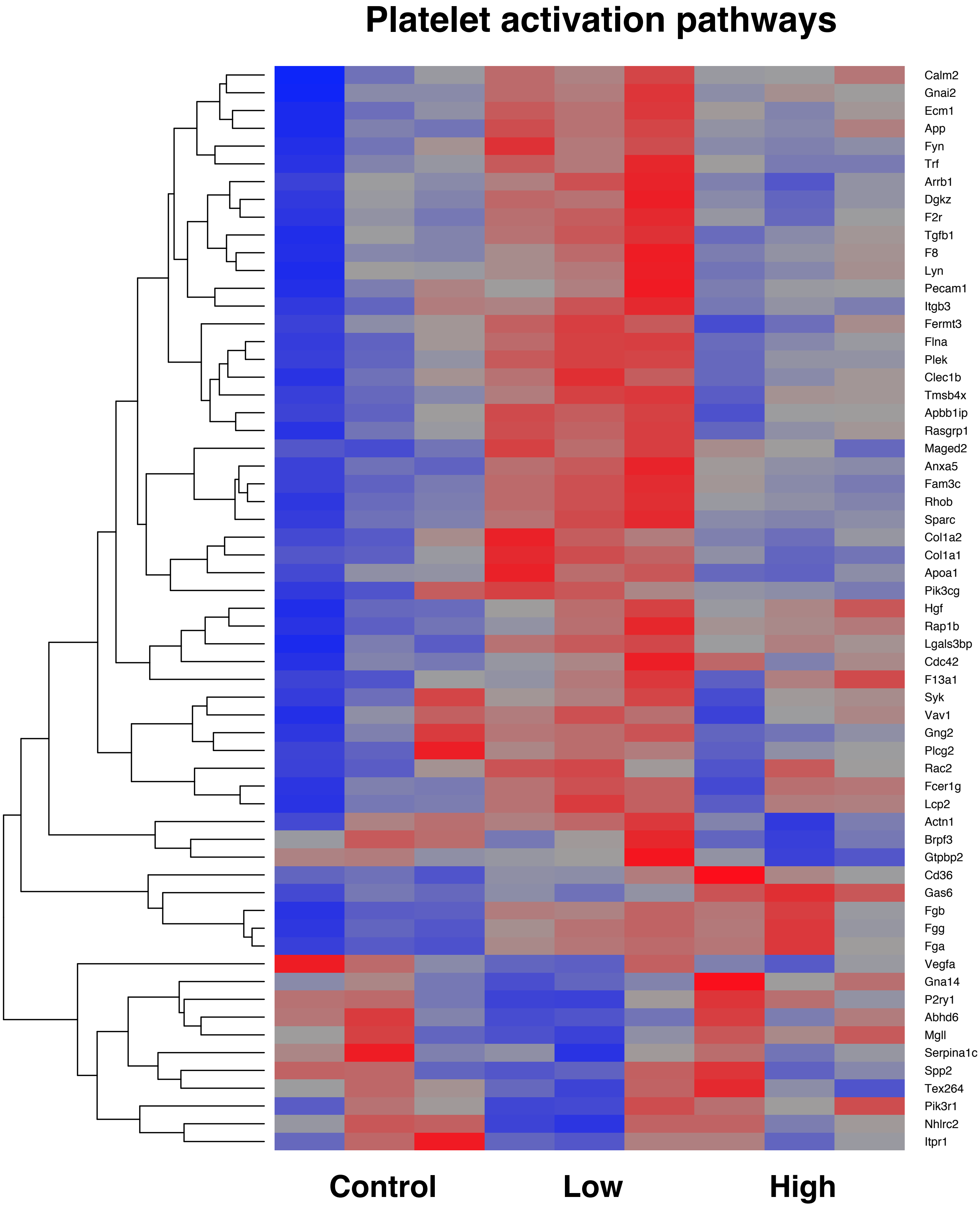

Supplementary Figure 10: RT-qPCR of selected drug-metabolizing P450s in livers of male and female ApoE3 and ApoE4 mice following exposure to vehicle, the low Cd dose (0.6 mg/L), and the high Cd dose (3mg/L). Data are expressed as % of the house keeping gene Gapdh. Asterisks: $p < 0.05$ (Analysis of variance).

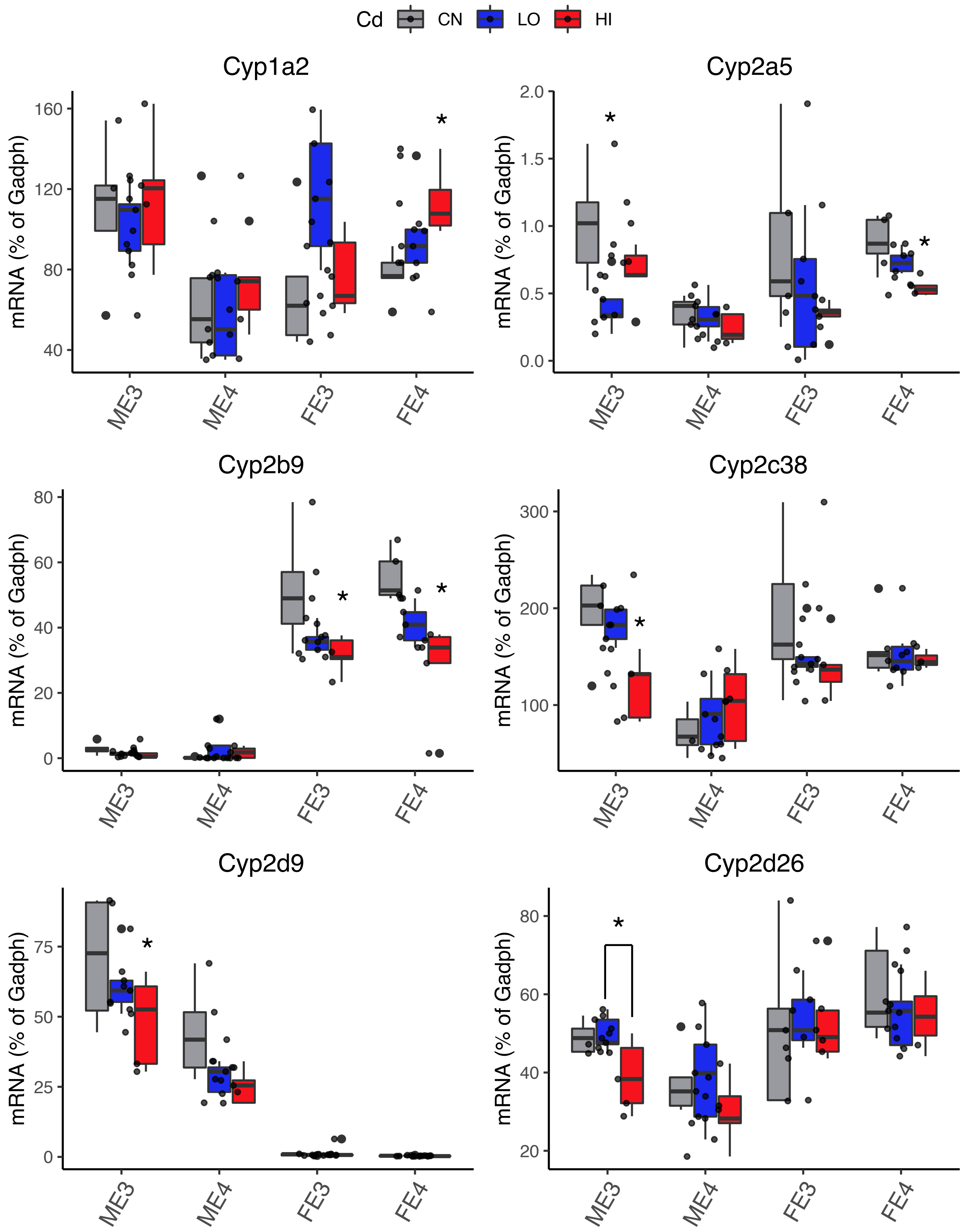

Supplemental Table 1. 16S rDNA Sequencing number of reads

Sample name	Sequence count	Filtered Reads	Percentage
E3ctrlF1	101338	41700	41.15%
E3ctrlF2	87800	39641	45.15%
E3ctrlF3	75223	34847	46.32%
E3ctrlF4	86096	36100	41.93%
E3ctrlF5	76426	36967	48.37%
E3ctrlM1	66728	27663	41.46%
E3ctrlM2	73343	33020	45.02%
E3ctrlM3	70288	31666	45.05%
E3ctrlM4	73979	35288	47.70%
E3ctrlM5	91720	46034	50.19%
E3highCdF1	88206	37594	42.62%
E3highCdF2	83125	33797	40.66%
E3highCdF3	94844	39724	41.88%
E3highCdF4	96506	42635	44.18%
E3highCdM1	90610	40357	44.54%
E3highCdM2	98164	47072	47.95%
E3highCdM3	102567	45899	44.75%
E3highCdM4	79314	40214	50.70%
E3lowCdF1	80464	35214	43.76%
E3lowCdF2	91590	35975	39.28%
E3lowCdF3	105193	46316	44.03%
E3lowCdF4	91726	41862	45.64%
E3lowCdM1	82458	38218	46.35%
E3lowCdM2	100912	54479	53.99%
E3lowCdM3	102459	50402	49.19%
E3lowCdM4	94302	46813	49.64%
E3lowCdM5	104929	48455	46.18%
E4ctrlF1	104020	48854	46.97%
E4ctrlF2	89413	42177	47.17%
E4ctrlF3	93929	41995	44.71%
E4ctrlF4	83787	37268	44.48%
E4ctrlF5	79121	37806	47.78%
E4ctrlM1	103093	46523	45.13%
E4ctrlM2	86603	41755	48.21%
E4ctrlM3	89481	45910	51.31%
E4ctrlM4	88054	44375	50.40%
E4highCdF1	80142	35780	44.65%
E4highCdF2	68560	27347	39.89%
E4highCdF3	78915	33229	42.11%
E4highCdF4	67797	35704	52.66%
E4highCdF5	74874	32588	43.52%
E4highCdM1	84784	39843	46.99%
E4highCdM2	76593	34206	44.66%

E4highCdM3	72337	34037	47.05%
E4highCdM4	88582	42943	48.48%
E4lowCdF1	76196	36353	47.71%
E4lowCdF2	99446	46110	46.37%
E4lowCdF3	78523	37255	47.44%
E4lowCdF4	74858	34521	46.12%
E4lowCdF5	77664	39603	50.99%
E4lowCdM1	83696	39371	47.04%
E4lowCdM2	71073	36016	50.67%
E4lowCdM3	80436	41651	51.78%
E4lowCdM4	86730	45629	52.61%

Supplemental Table 2. Liver Transcriptome Mapping Statistics

Sample ID	Total Reads	Mapped Reads	% Mapped	Sample Description
Julia_Cui_Cd_1.206654.bam	59399200	45324706	76.31%	M_ApoE3_CN#1
Julia_Cui_Cd_2.206655.bam	58421863	46655850	79.86%	M_ApoE3_CN#2
Julia_Cui_Cd_3.206656.bam	62206667	47986255	77.14%	M_ApoE3_CN#3
Julia_Cui_Cd_4.206657.bam	52222652	41331591	79.14%	M_ApoE3_LowCd#1
Julia_Cui_Cd_5.206658.bam	51301620	40421201	78.79%	M_ApoE3_LowCd#2
Julia_Cui_Cd_6.206659.bam	52897169	40990671	77.49%	M_ApoE3_LowCd#3
Julia_Cui_Cd_7.206660.bam	49328760	41278096	83.68%	M_ApoE3_HighCd#1
Julia_Cui_Cd_8.206661.bam	50559772	39148651	77.43%	M_ApoE3_HighCd#2
Julia_Cui_Cd_9.206662.bam	50893219	39525687	77.66%	M_ApoE3_HighCd#3
Julia_Cui_Cd_10.206663.bam	53873140	40553937	75.28%	M_ApoE4_CN#1
Julia_Cui_Cd_11.206664.bam	54651061	42432145	77.64%	M_ApoE4_CN#2
Julia_Cui_Cd_12.206665.bam	50602146	40672873	80.38%	M_ApoE4_CN#3
Julia_Cui_Cd_13.206666.bam	54705591	41435250	75.74%	M_ApoE4_LowCd#1
Julia_Cui_Cd_14.206667.bam	46187304	38768045	83.94%	M_ApoE4_LowCd#2
Julia_Cui_Cd_15.206668.bam	57817440	48853358	84.50%	M_ApoE4_LowCd#3
Julia_Cui_Cd_16.206669.bam	59113384	48583563	82.19%	M_ApoE4_HighCd#1
Julia_Cui_Cd_17.206670.bam	47595795	38533693	80.96%	M_ApoE4_HighCd#2
Julia_Cui_Cd_18.206671.bam	48826745	39704493	81.32%	M_ApoE4_HighCd#3
Julia_Cui_Cd_19.206672.bam	46828853	37499943	80.08%	F_ApoE3_CN#1
Julia_Cui_Cd_20.206673.bam	47650533	38140024	80.04%	F_ApoE3_CN#2
Julia_Cui_Cd_21.206674.bam	48012018	38361765	79.90%	F_ApoE3_CN#3
Julia_Cui_Cd_22.206675.bam	44524845	36306827	81.54%	F_ApoE3_LowCd#1
Julia_Cui_Cd_23.206676.bam	48538288	39901403	82.21%	F_ApoE3_LowCd#2
Julia_Cui_Cd_24.206677.bam	48372517	39329534	81.31%	F_ApoE3_LowCd#3
Julia_Cui_Cd_25.206678.bam	47543179	37643761	79.18%	F_ApoE3_HighCd#1
Julia_Cui_Cd_26.206679.bam	43221903	35851218	82.95%	F_ApoE3_HighCd#2
Julia_Cui_Cd_27.206680.bam	53566699	43905373	81.96%	F_ApoE3_HighCd#3
Julia_Cui_Cd_28.206681.bam	45460039	37297029	82.04%	F_ApoE4_CN#1
Julia_Cui_Cd_29.206682.bam	45481169	37482250	82.41%	F_ApoE4_CN#2
Julia_Cui_Cd_30.206683.bam	45928871	36355404	79.16%	F_ApoE4_CN#3
Julia_Cui_Cd_31.206684.bam	45049490	36575414	81.19%	F_ApoE4_LowCd#1
Julia_Cui_Cd_32.206685.bam	49413951	38334599	77.58%	F_ApoE4_LowCd#2
Julia_Cui_Cd_33.206686.bam	49642014	39706693	79.99%	F_ApoE4_LowCd#3
Julia_Cui_Cd_34.206687.bam	46772613	37290394	79.73%	F_ApoE4_HighCd#1
Julia_Cui_Cd_35.206688.bam	45484425	37286239	81.98%	F_ApoE4_HighCd#2
Julia_Cui_Cd_36.206689.bam	45599147	37048562	81.25%	F_ApoE4_HighCd#3
Julia_Cui_Cd_37.206690.bam	45658631	36942782	80.91%	ileum_F_ApoE3_CN#1
Julia_Cui_Cd_38.206691.bam	47569793	37945196	79.77%	ileum_F_ApoE3_CN#2
Julia_Cui_Cd_39.206692.bam	43911979	35754791	81.42%	ileum_F_ApoE3_CN#3
Julia_Cui_Cd_40.206693.bam	44792530	36684213	81.90%	ileum_F_ApoE3_HighCd#1
Julia_Cui_Cd_41.206694.bam	46118901	36840913	79.88%	ileum_F_ApoE3_HighCd#2
Julia_Cui_Cd_42.206695.bam	60823368	40452176	66.51%	ileum_F_ApoE3_HighCd#3
Julia_Cui_Cd_43.206696.bam	44121039	35670834	80.85%	ileum_F_ApoE4_CN#1
Julia_Cui_Cd_44.206697.bam	44527260	36452308	81.87%	ileum_F_ApoE4_CN#2
Julia_Cui_Cd_45.206698.bam	43873037	36186055	82.48%	ileum_F_ApoE4_CN#3
Julia_Cui_Cd_46.206699.bam	46680897	36277502	77.71%	ileum_F_ApoE4_HighCd#1
Julia_Cui_Cd_47.206700.bam	45393600	35860368	79.00%	ileum_F_ApoE4_HighCd#2
Julia_Cui_Cd_48.206701.bam	45206738	36521566	80.79%	ileum_F_ApoE4_HighCd#3

Supplemental Table 3: Comparison of the cadmium effect on the liver transcriptome and the LINCS databa

Sex	Genotype	Dose	Direction	Chemical	Adjusted P-value	Combined Score
Male	ApoE3	Low	Up	QL-XII-47	0.001	140.13
				BRD-K92317137	0.019	238.53
				GSK-2126458	0.029	195.48
				SB-203580	0.032	135.01
				Mitoxantrone	0.039	122.48
			Down	GDC-0941	$1.09 * 10^{-4}$	266.01
				Selumetinib	0.002	179.14
				AZD-7762	0.001	176.53
				Radicicol	0.002	119.3
				GSK-2126458	0.002	149.37
		High	Up			
			Down	GSK-2126458	0.015	218.34
				KU-60019	0.022	170.65
				INK-128	0.022	171.52
				JW-7-24-1	0.024	173.09
				Torin-2	0.025	462.24
	ApoE4	Low	Up	Withaferin-a	0.004	115.64
				Radicicol	0.007	104
				AT-7519	0.025	121.026
				QL-XII-47	0.024	97.43
				CP466722	0.036	123.53
			Down	HG-5-113-01	$2.03 * 10^{-4}$	78.34
				Ropinirole HCl	$3.63 * 10^{-4}$	64.55
				BMS-387032	$3.63 * 10^{-4}$	64.55
				CX-5461	$7.42 * 10^{-4}$	69.78
				V-ama37	$9.76 * 10^{-4}$	103.67
		High	Up	Pelitinib	$5.59 * 10^{-15}$	244.82
				QL-X-138	$7.61 * 10^{-13}$	168.18
				Vorinostat	$1.30 * 10^{-12}$	179.6
				Afatinib	$1.23 * 10^{-11}$	145.55
				CHIR-99021	$1.95 * 10^{-11}$	157.42
			Down			
				WYE-125132	$2.17 * 10^{-4}$	622.59

Supplemental Table 3: Comparison of the cadmium effect on the liver transcriptome and the LINCS database.

Sex	Genotype	Dose	Direction	Chemical	Adjusted P-value	Combined Score
Male	ApoE3	Low	Up	QL-XII-47	0.001	140.13
				BRD-K92317137	0.019	238.53
				GSK-2126458	0.029	195.48
				SB-203580	0.032	135.01
				Mitoxantrone	0.039	122.48
			Down	GDC-0941	$1.09 * 10^{-4}$	266.01
				Selumetinib	0.002	179.14
				AZD-7762	0.001	176.53
				Radicicol	0.002	119.3
				GSK-2126458	0.002	149.37
		High	Up			
			Down	GSK-2126458	0.015	218.34
				KU-60019	0.022	170.65
				INK-128	0.022	171.52
				JW-7-24-1	0.024	173.09
				Torin-2	0.025	462.24
	ApoE4	Low	Up	Withaferin-a	0.004	115.64
				Radicicol	0.007	104
				AT-7519	0.025	121.026
				QL-XII-47	0.024	97.43
				CP466722	0.036	123.53
			Down	HG-5-113-01	$2.03 * 10^{-4}$	78.34
				Ropinirole HCl	$3.63 * 10^{-4}$	64.55
				BMS-387032	$3.63 * 10^{-4}$	64.55
				CX-5461	$7.42 * 10^{-4}$	69.78
				V-ama37	$9.76 * 10^{-4}$	103.67
		High	Up	Pelitinib	$5.59 * 10^{-15}$	244.82
				QL-X-138	$7.61 * 10^{-13}$	168.18
				Vorinostat	$1.30 * 10^{-12}$	179.6
				Afatinib	$1.23 * 10^{-11}$	145.55
				CHIR-99021	$1.95 * 10^{-11}$	157.42
			Down			
				WYE-125132	$2.17 * 10^{-4}$	622.59

Female	ApoE3	Low	Up	QL-X-138	$1.33 * 10^{-4}$	437.91
				AZD-8055	$7.51 * 10^{-3}$	525.03
				OSI-027	0.004	355.85
				Dasatinib	0.01	482.16
			Down			
		High	Up	Alvocidib	$8.51 * 10^{-4}$	164.53
				Dinaciclib	0.003	346.27
				GSK-690693	0.004	142.21
				PF-477736	0.016	125.01
				Celastrol	0.021	96.01
			Down	Mocetinostat	$4.82 * 10^{-11}$	253.67
				Torin-2	$1.16 * 10^{-10}$	229.37
				PD-0325901	$6.54 * 10^{-10}$	237.45
				Pracinostat	$4.17 * 10^{-9}$	164.52
				Torin-1	$1.39 * 10^{-8}$	116.14
	ApoE4	Low	Up	AS-605240	0.035	376.82
				CGP-60474	0.037	213.54
				PD-0325901	0.032	339.82
				Stock1s-53863	0.043	371.14
				Gefitinib	0.044	462.77
			Down			
		High	Up			
			Down	Geldanamycin	0.024	230.26
				NVP-AUY922	0.036	314.79

Supplemental Table 4 PCR primer sequences for inflammation genes, universal bacteria, A. muciniphila and CYP genes

Targets	Primers (F: forward; R: reverse)	Sequence (5'-3')
IL-1b	mIL1B-F	CAGGCAGGCAGTATCACTCA
Il-1b	mIL1B-R	AGGTGCTCATGTCCTCATCC
TNFa	mTNFa-F	ACGGCATGGATCTCAAAGAC
TNFa	mTNFa-R	GTGGGTGAGGAGCACGTAGT
IL-6	mIL6-F	CCGGAGAGGAGACTTCACAG
IL-6	mIL6-R	TCCACGATTTCCCAGAGAAC
Universal bacteria	GTGSTGCAYGGYTGTCGTCA	ACGTCRTCCMCACCTTCCTC
Akkermansia muciniphila	ATCATTCACAATGGGGGAAA	CCCACATGACAGGGGTTTAC
Cyp1a2	GACATGGCCTAACGTGCAG	GGTCAGAAAGCCGTGGTTG
Cyp2a5	AGTGCATGGGAAGGGGAAGAGAGT	ACCCAAGGCCAAAGCACACAGA
Cyp2b9	GCATCACAGCCAACATCATC	TCGAGGAGTTCCTGCTGTTT
Cyp2c38	CTACCCCATGCAGTGACCTG	TGGCCAGGGTCAAACATCTC
Cyp2d9	AAGGCTGGCTGACAAGGCCC	TCGGGGTGCTTGGACAGGGT
Cyp2d26	GCCATCTTCCTGCTTCTGGT	TGTTCTCGAAGTCCACCTGC
