# Supplementary material for: Cadmium exposure modulates the gut-liver axis in an Alzheimer’s disease mouse model
Source: Commun Biol. 2021 Dec 15;4:1398. doi: 10.1038/s42003-021-02898-1 (PMC8674298; doi:10.1038/s42003-021-02898-1)
Supplement: Supplementary file 2 — Description of Additional Supplementary Files [file 42003_2021_2898_MOESM2_ESM.pdf]

Description of Additional Supplementary Files

File name: Supplementary Data 1

Description: GC-MS quantification of short chain and medium chain.
